# Antidepressant-like activity, active components and related mechanism of *Hemerocallis citrina* Baroni extracts

**DOI:** 10.3389/fphar.2022.967670

**Published:** 2022-08-29

**Authors:** Jinghong Liu, Tian Ye, Shuaiyong Yang, Xiaohong Zhong, Wei He, Mengtao Xu, Jinpeng Fang, Miao Deng, Ning Xu, Jianguo Zeng, Zhixing Qing

**Affiliations:** ^1^ College of Horticulture, Hunan Agricultural University, Changsha, China; ^2^ Department of Bioengineering and Environmental Science, Changsha University, Changsha, China; ^3^ Hunan Key Laboratory of Traditional Chinese Veterinary Medicine, Hunan Agricultural University, Changsha, China; ^4^ Green Melody Bioengineering Group Company Limited, Changsha, China; ^5^ College of Food Science and Technology, Hunan Agricultural University, Changsha, China; ^6^ Datong Daylily Industrial Development Research Institute, Datong, China

**Keywords:** chemical constituents, HPLC-Q-TOF-MS, CUMS mice, hemerocallis citrina baroni [asphodelaceae], antidepressant-like effect, intestinal flora

## Abstract

*Hemerocallis citrina* Baroni [Asphodelaceae], which is traditional herbal medicine, has been widely used for treating depressive disorders in Eastern-Asia countries. However, the active compounds and corresponding mechanism of anti-depression are not yet completely clarified. In this study, the anti-depressive activities of six *H. citrina* extracts were primarily evaluated. The results showed that the water extract of *H. citrina* flowers (HCW) displays significant anti-depressive activity. A total of 32 metabolites were identified from HCW by high-performance liquid chromatography/quadrupole time-of-flight mass spectrometry (HPLC-Q-TOF-MS) and nuclear magnetic resonance (NMR). And then, the anti-depressive activity of the high-level compound (rutin) in HCW was also estimated. The results indicated that rutin displayed significant anti-depressive activity and was one of the main active ingredients. Finally, the anti-depressive mechanisms of HCW and rutin were investigated based on the intestinal microorganisms. The results showed that HCW and rutin increase the diversity and richness of the intestinal flora and regulate the specific intestinal microorganisms such as *Bacteroides* and *Desulfovibrio* genera in depressed mice. This work marks the first comprehensive study of the active components, anti-depressive activities and corresponding mechanisms of different *H. citrina* extracts, which provide a potential possibility for developing new antidepressants.

## Graphical Abstract

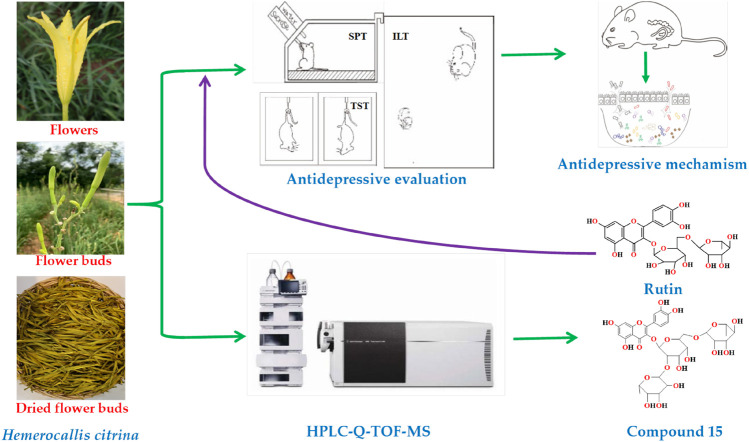

## 1 Introduction

Depression, a mental disease with high morbidity and mortality, has become a severe public health problem in the 21st century. According to the prediction by the World Health Organization, depression will become the disease with the heaviest economic burden in the world by 2030 (World Health Organization, 2017; Miller and Campo, 2021). At present, synthesized drugs are the most commonly used and effective treatment means in clinical traits, which have disadvantages such as low effective rate, serious side effects, and high price (Krishnan and Nestler, 2008; Carhart-Harris et al., 2021). However, traditional herbal medicines have unique advantages in preventing and treating depression as alternative and complementary therapies. Therefore, the development of new antidepressants from traditional herbal medicines has been researched hot.

*H. citrina* Baroni (It was also called “Huang Hua Cai” in Chinese) has been widely grown in China, Japan, and Korea, and its flower buds are one of the most commonly consumed vegetables in Asia (Ma et al., 2018; Liu et al., 2020). The flower buds of *H. citrina* have been recorded to relieve depression in the medicinal book “Compendium of Materia Medica”, which is a famous Chinese encyclopedia of medicine written by Li et al. (2017) in the Ming dynasty (Xu et al., 2020; Qing et al., 2021a). Modern pharmacology has also proved that flower buds of *H. citrina* extract have significant antidepressant activity, and the polyphenols and flavonoids were regarded as the main active components (Lin et al., 2013; Xu et al., 2016). However, the specific active ingredients in the extract of *H. citrina*, which may display prominent antidepressant-like activity, were rarely identified and needed further investigation.

The active constituents undergo successive changes during plant growth, and metabolites vary in fresh and dry flower buds of *H. citrina* (Qing et al., 2017a; Yu et al., 2020; Qing et al., 2021b). In some previous studies (Du et al., 2014; Xu et al., 2016), a chronic unpredictable mild stress (CUMS) model was used to evaluate the anti-depressive effect of *H. citrina* that only took one type of the flower buds (mainly using the dried flower buds of *H. citrina* as the materials), which may cause a series of active constituents to be neglected and not estimated. In the present study, we comprehensively evaluate the antidepressant-like activities of extracts produced by dried flower buds, fresh flower buds, and flowers of *H. citrina* (Supplementary Figure S1)*.*

The antidepressant activity of *H. citrina*. extracts and related mechanisms have been investigated in previous studies. *H. citrina* extracts could increase the levels of monoamine neurotransmitters, such as 5-hydroxytryptamine (5-HT), dopamine (DA) and norepinephrine (NE), in the brain of depressed mice (monoamine hypothesis) (Gu et al., 2012; Lin et al., 2013; Xu et al., 2016; Qing et al., 2021a). In addition, the extracts of *H. citrina* were able to increase the content of BDNF (neurotrophic hypothesis) (Yi et al., 2012) and reduce IL-1β, IL-6, TNF-α and malondialdehyde (MDA) levels (stress hypothesis) (Liu et al., 2014) in the brain of depressed mice. With the development of human health and gut microbes, depression is inextricably linked with changes in intestinal microorganisms. However, the relationships between the antidepressant-like activity of *H. citrina* extracts and intestinal microorganism variations were rarely studied and need further investigation.

In this study, the antidepressant-like activities of 6 different extracts from *H. citrina* were primarily assessed using a CUMS model. And then, the main chemical constituents of active extract were identified by HPLC-Q-TOF-MS and NMR technology. Finally, the mechanisms of antidepressant-like activity were investigated based on the intestinal flora.

## 2 Results and discussion

### 2.1 The anti-depressive activities of the extracts of *H. citrina* flowers and fresh flower buds

**FIGURE 1:**
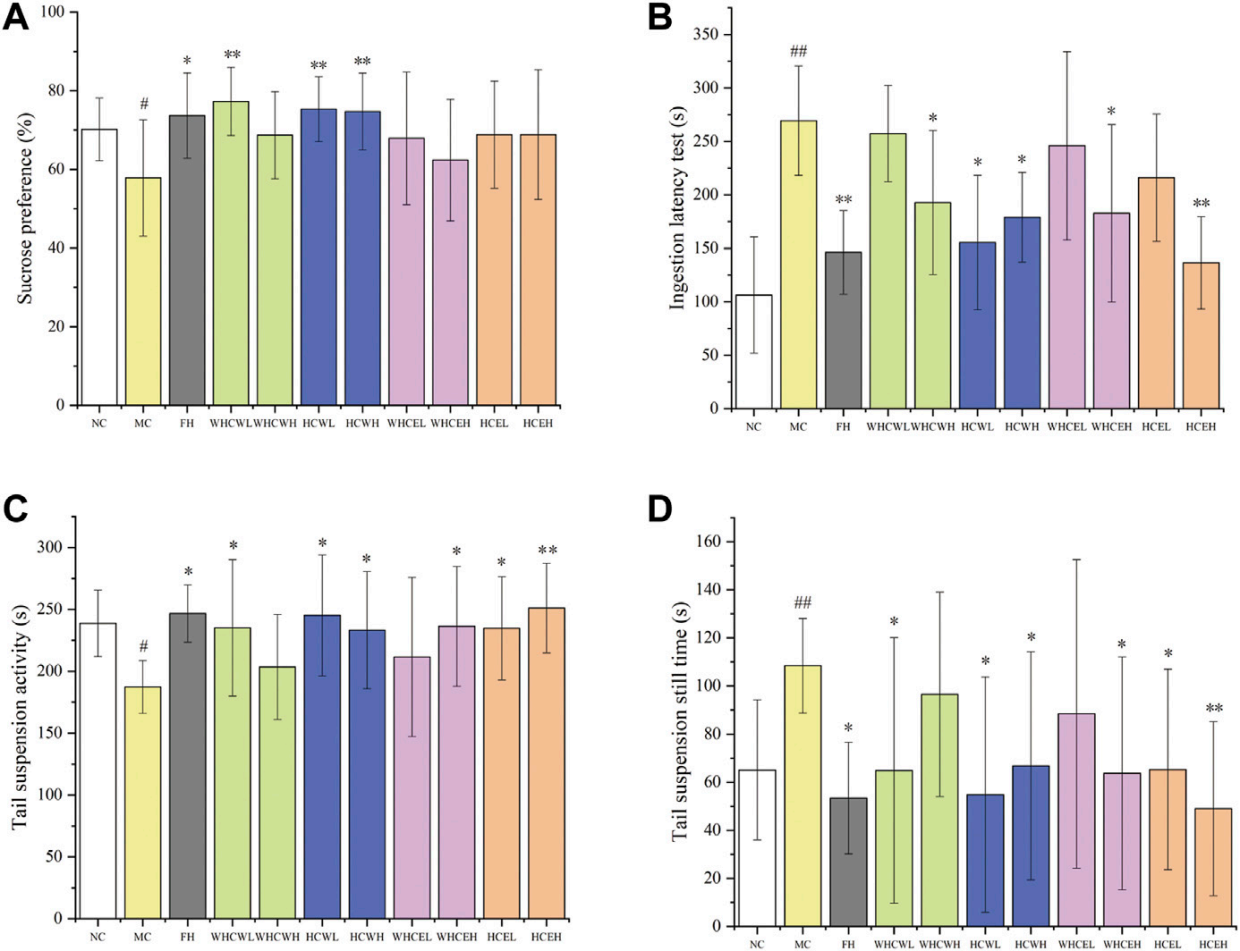
The effects of different dose extracts of *H. citrina* flowers and fresh flower buds on the behaviors of CUMS mice. **(A)** Sucrose preference test, **(B)** ingestion latency test, **(C)** tail suspension activity test, and **(D)** tail suspension still time test. Data are reported as mean ± SD. For statistical significant, ^#^ *p* < 0.05, ^##^ *p* < 0.01 compared with the normal control group; *p < 0.05, **p < 0.01 compared with the model control group. NC, normal group; MC, model group; FH, Fluoxetine hydrochloride group; WHCWL and WHCWH, low and high-dose of water extracts of fresh flower buds; HCWL and HCWH, low and high-dose of water extracts of flowers; WHCEL and WHCEH, low and high-dose of 80% ethanol extracts of fresh flower buds; HCEL and HCEH, low and high-dose of 80% ethanol extracts of flowers; low-dose: 200 mg/kg; high-dose, 500 mg/kg.

To evaluate the anti-depressive activities of flowers and fresh flower buds (Supplementary Figure S1), the water and 80% ethanol extracts of both parts (a total of 4 different extracts with low and high-dose, namely WHCWL, WHCWH, HCWL, HCWH, WHCEL, WHCEH, HCEL, and HCEH) were employed. The result showed that the Sucrose preference test (SPT) of the model control group was significantly lower (*p* < 0.05) compared with the normal control group, which indicated that the CUMS mouse model was successfully established (Figure 1A). The SPT index of different dose HCW groups (HCWL and HCWH), WHCW low-dose group (WHCWL) and fluoxetine hydrochloride (FH) group was significantly increased compared to that of the model group (*p* < 0.01 or *p* < 0.05). According to the SPT index, the HCW displayed stronger anti-depressive activity than the positive control in the corresponding dose.

Compared with the normal control group, the Ingestion latency test (ILT) of mice in the model control group was significantly prolonged (*p* < 0.01), however, the ILT index in the FH group was significantly shorter than that in the model group (*p* < 0.01) (Figure 1B). The ILT of the depressed mice in low and high-dose HCW groups (HCWL and HCWH) was significantly decreased (*p* < 0.05) compared to the model control group, and the high-dose HCW group had a more shorted ILT index than that of the low-dose group. In addition, The ILT of the depressed mice in the high-dose WHCW (WHCWH), WHCE (WHCEH), and HCE (HCEH) were significantly decreased (*p* < 0.05, or *p* < 0.01) compared to the model control group (Figure 1B).

Compared with the normal control group, the activity time of the model control group was significantly decreased (*p* < 0.05), and the resting time was significantly prolonged (*p* < 0.01), which indicated that the CUMS mouse model was successfully established (Figures 1C,D). Compared with the model group, the activity time was significantly prolonged (*p* < 0.05) and the resting time was significantly decreased (*p* < 0.05) in low and high-dose groups of HCW. The low-dose WHCW (WHCWL), high-dose WHCE (WHCWH), low and high-dose WHCE (WHCEL and WHCEH), and FH groups display similar anti-depressive activities with the HCW groups (Figures 1C,D).

The results of SPT, ILT, and Tail suspension test (TST) experiments indicated that the low and high-dose HCW groups display significant anti-depressive activity, and the water extracts of flowerings (HCWL and HCWH) showed better activities than that of ethanol extracts (HCEL and HCEH). In addition, the extracts of flowerings (HCWL, HCWH, HCEL, and HCEH) display more significant anti-depressive activities than that of flower buds (WHCWL, WHCWH, WHCEL, and WHCEH).

### 2.2 The anti-depressive activities of the extracts of *H. citrina* flowers and dry flower buds

**FIGURE 2:**
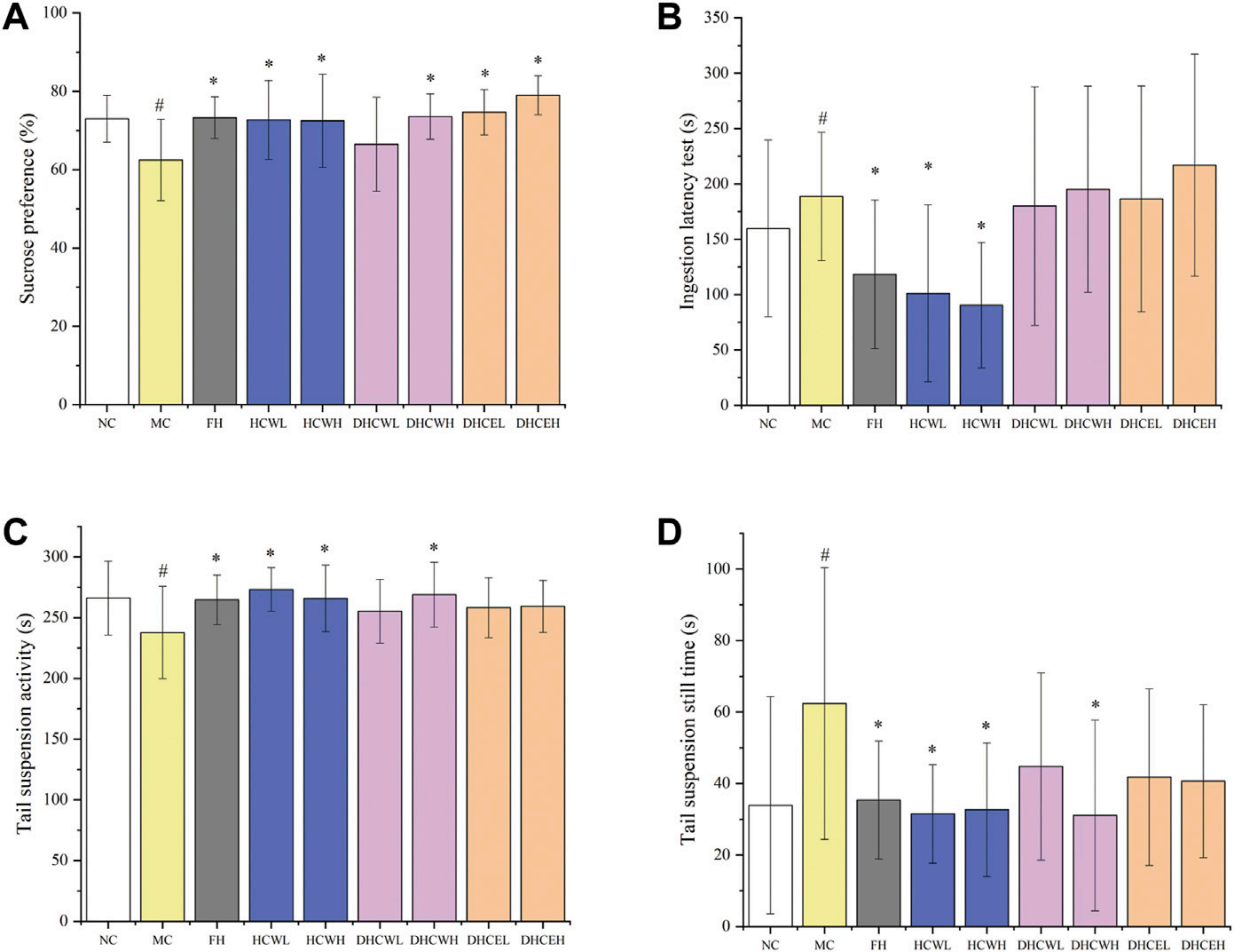
The effects of different dose extracts of *H. citrina* flowers and dried flower buds on the behaviors of CUMS mice. **(A)** Sucrose preference test, **(B)** ingestion latency test, **(C)** tail suspension activity test, and **(D)** tail suspension still time test. Data are reported as mean ± SD. For statistical significant, ^#^ *p* < 0.05, ^##^ *p* < 0.01 compared with the normal control group; *p < 0.05, **p < 0.01 compared with the model control group. NC, normal group; MC, model group; FH, Fluoxetine hydrochloride group; HCWL and HCWH, low and high-dose of water extracts of flowers; DHCWL and DHCWH, low and high-dose of water extracts of dried flower buds; DHCEL and DHCEH: low and high-dose of 80% ethanol extracts of dried flower buds; low-dose, 200 mg/kg; high-dose, 500 mg/kg.

To evaluate the anti-depressive activities of flowers and dry flower buds of *H. citrina* (Supplementary Figure S1), the water and 80% ethanol extracts of both samples (a total of 3 different extracts with low and high-dose, namely HCWL, HCWH, DHCWL, DHCWH, DHCEL, and DHCEH) were employed. The result showed that the SPT of the model control group was significantly lower (*p* < 0.05) compared with the normal control group, which indicated that the CUMS mouse model was successfully established (Figure 2A). Compared to the model group, the SPT of low and high-dose HCW and DHCE groups (HCWL, HCWH, DHCWL, and DHCWH), high-dose DHCW group (DHCWH) and FH group was significantly increased (*p* < 0.05).

As shown in Figure 2B, the ILT of mice in the model control group was significantly prolonged (*p* < 0.05) compared to the normal control group, which also indicated that the CUMS mouse model was successfully established. The ILT index of mice in low and high-dose HCW groups (HCWL and HCWH) and the positive control group (FH) were significantly decreased (*p* < 0.05) compared with the model control group. However, the ILT of mice in the other four groups (DCWL, DCWH, DHCEL, and DHCEH) were not decreased significantly.

Compared with the normal control group, the activity time of the model control group was significantly decreased (*p* < 0.05), and the resting time was significantly prolonged (*p* < 0.05), both results showed that the CUMS mouse model was successfully established (Figures 2C,D). The activity time of the depressed mice in the FH group, the low and high-dose group of HCW (HCWL and HCWH), and the high-dose DHCW group (DHCWH) were significantly prolonged (*p* < 0.05), and the resting time was decreased significantly (*p* < 0.05) (Figures 2C,D) compared with the model group.

The results of SPT, ILT and TST in the second anti-depressive experiment indicated that the low and high-dose HCW groups (HCWL and HCWH) also display significant anti-depressive activity. However, the water and 80% ethanol extracts of *H. citrina* dry flower buds (DHCWL, DHCWH, DHCEL, and DHCEH) did not show noteworthy anti-depressive activities. In summary, HCW displayed significant anti-depressive activity in the twice anti-depressive experiments, and the extracts of flowers showed more potent anti-depressive activity than that of fresh and dry flower buds.

### 2.3 Analysis and isolation of primary metabolites from *H. citrina* flowers

**FIGURE 3:**
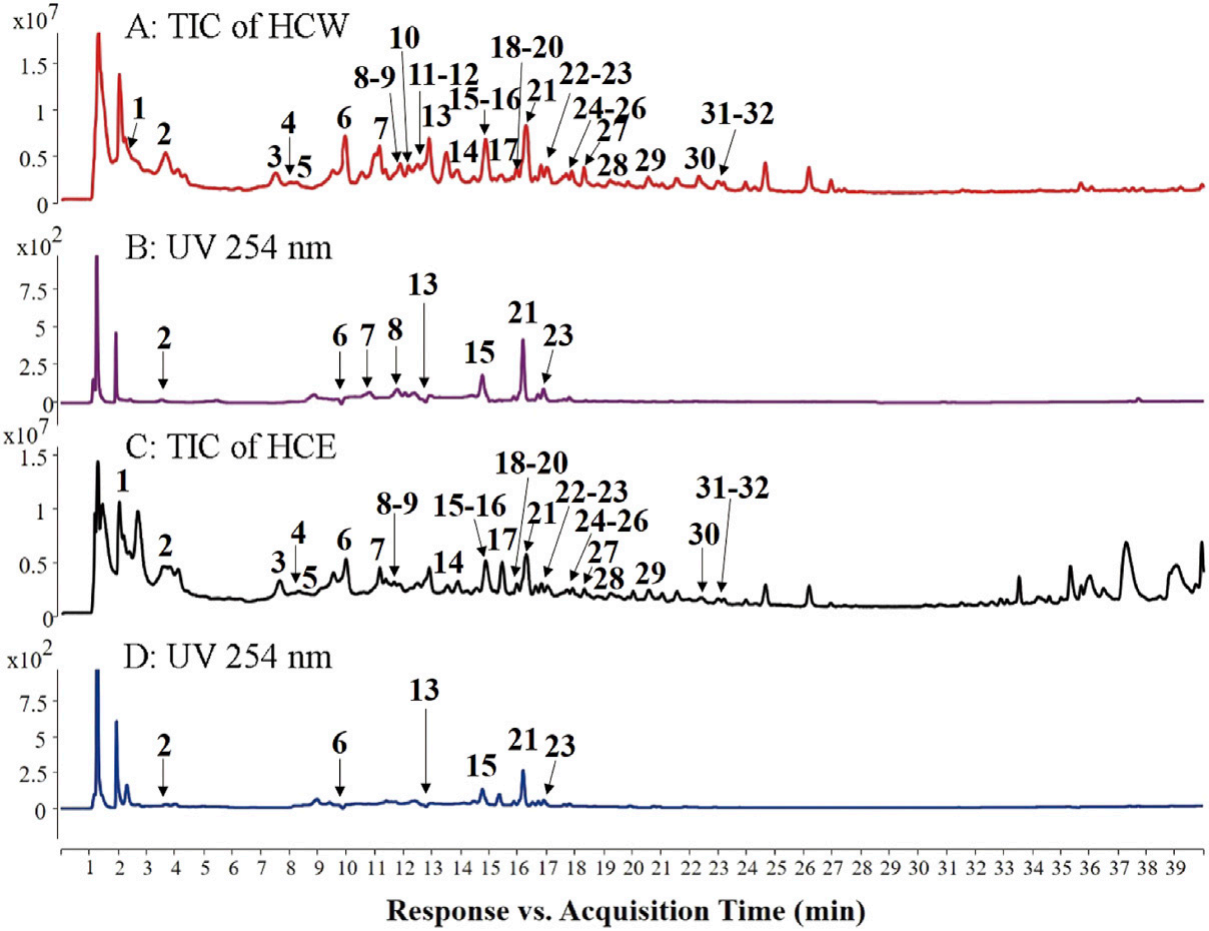
Total ion chromatograms (TICs, **(A)** and **(C)** in ESI^−^ mode and UV chromatograms (254 nm, **(B)** and **(D)** of HCW and HCE.

The HCW and HCE of *H. citrina* was preliminarily analyzed by HPLC-Q-TOF-MS to find the main ingredients contributing to the antidepressant activity. A total of 32 high-level metabolites, including 18 flavonoids, 7 chlorogenic acid-type, 3 polyphenols, 2 acetamide alkaloids and 2 diterpenoid saponins, were screened and tentatively identified by their tandem mass spectrometry (MS/MS) [(Table 1 and Figure 3) (Liu et al., 2020)]. And then, the high-content ingredient (compound **15**) was further isolated by the MS-guided isolation method (Qing et al., 2014; Qing et al., 2016; Qing et al., 2017b), and its structure was unambiguously identified by nuclear magnetic resonance (NMR) data. The specific structural identification of the primary metabolites by HPLC-Q-TOF-MS and NMR is as follows:

**TABLE 1:** Primary metabolites identified in HCW and HCE by HPLC-Q-TOF-MS.

No.	*t*_R_ (min.)	[M-H]^-^(*m/z*)	Error (ppm)	Molecular formula	MS/MS fragment ions (*m/z*)	Tentative identification
1	2.83	243.0991	2.7	C_10_H_16_N_2_O_5_	225.0891, 208.0696, 197.0921, 181.0971, 164.0689, 144.0296	Pinnatanine A
2	3.68	353.0878	1.8	C_16_H_18_O_9_	191.0553, 179.0331, 135.0448	5-Caffeoylquinic acid
3	7.57	353.0881	1.9	C_16_H_18_O_9_	191.0554, 179.0342, 135.0446	4-Caffeoylquinic acid
4	7.96	337.0938	3.3	C_16_H_18_O_8_	191.0534, 173.0472, 163.0377	Dehydroxy-chlorogenic acid Ⅱ
5	8.27	231.1346	−0.4	C_10_H_20_N_2_O_4_	213.1249, 195.1153, 185.1296, 168.1036, 144.0299	1′,2′,3′,4′-Tetrahydro-5′-deoxy-pinnatanine
6	9.92	337.0934	3.1	C_16_H_18_O_8_	191.0571, 173.0405, 163.0382	Dehydroxy-chlorogenic acid Ⅰ
7^a^	11.13	353.0885	1.7	C_16_H_18_O_9_	191.0544, 179.0325, 135.0431	Chlorogenic acid
8	11.88	337.0925	0.1	C_16_H_18_O_8_	191.0556, 163.0404, 119.0509	Dehydroxy-chlorogenic acid
9^a^	11.88	179.0344	−0.4	C_9_H_8_O_4_	135.0445, 117.0355, 107.0494	Caffeic acid
10	12.19	555.1723	1.3	C_25_H_32_O_14_	231.0658, 216.0385, 179.0589	3-Ethoxy-vanillic acid-*O*-glucoside
11	12.51	431.1924	1.1	C_19_H_30_O_8_	223.1323, 205.1222, 153.0919	Roseoside
12	12.51	387.1631	−0.2	C_19_H_32_O_8_	225.0770, 207.0652	Phlomuroside
13	12.90	337.0931	1.3	C_16_H_18_O_8_	191.0546, 173.0431, 163.0391	Dehydroxy-chlorogenic acid Ⅲ
14	13.85	771.1987	−0.6	C_33_H_40_O_21_	609.1443, 462.0791, 301.0329	Rutin-*O*-glucoside
15^a^	14.86	755.2028	−1.2	C_33_H_40_O_20_	591.1280, 301.0243, 300.0173, 271.0139, 178.9875, 150.9928	Quercetin 3-*O*-*α*-*L*-rhamnopyranosyl-(1→6) -[*α*-*L*-rhamnopyranosyl-(1→2)]-*β*-*D*-glucopyranoside
16	14.86	625.1385	−3.2	C_27_H_30_O_17_	317.0193, 316.0125, 178.9879	Myricetin-*O*-rutinoseide
17	15.45	479.0827	−0.3	C_21_H_20_O_13_	316.0154, 287.0080, 271.0181	Myricetin-*O*-glucoside
18	15.96	739.2097	1.6	C_33_H_40_O_19_	593.1559, 285.0398, 284.0317 255.0293, 178.9977, 151.0053	Kaempferol-*O*-rhamnoside-*O*-rutinoseide
19	15.96	769.2195	0.2	C_34_H_42_O_20_	315.0492, 314.0439, 299.0186, 178.9999, 150.9997	Isorhamnetin-*O*-rhamnoside-*O*-rutinoseide
20	15.96	449.0718	0.2	C_20_H_18_O_12_	316.0211, 287.0174, 271.0224, 178.9985, 151.0012	Myricetin-*O*-arabinose
21^a^	16.27	609.1460	0.8	C_27_H_30_O_16_	301.0336, 300.0265, 271.0214, 178.9985, 151.0023	Rutin
22	16.82	463.0877	−0.2	C_21_H_20_O_12_	317.0285, 316.0219, 287.0179, 271.0233, 178.9975, 151.0004	Myricetin-*O′*-rhamnoside
23	17.06	579.1347	−0.3	C_26_H_28_O_15_	433.0719, 301.0340, 300.0274, 271.0241, 178.9985, 151.0037	Quercetin-*O*-arabinose-*O′*-rhamnoside
24	17.73	315.0504	1.8	C_16_H_12_O_7_	300.0281, 151.9991	Isorhamnetin
25	17.73	433.0769	−1.1	C_20_H_18_O_11_	301.0352, 300.0271, 271.0239, 255.0305, 178.9967, 151.0035	Quercetin-*O*-arabinose
26	17.92	623.1619	0.8	C_28_H_32_O_16_	315.0501, 314.0454, 151.0015	Isorhamnetin-rutinoseide
27	18.31	563.1419	2.7	C_26_H_28_O_14_	285.0392, 284.0318, 255.0307, 227.0375, 151.0041	Kaempferol-*O*-rhamnoside-*O′*-arabinose
28	18.79	593.1507	−0.3	C_27_H_30_O_15_	314.0423, 299.0199, 271.0242	Isorhamnetin-*O*-rhamnoside-*O′*-arabinose
29	19.88	507.1133	−0.7	C_23_H_24_O_13_	344.0544, 316.0510, 301.0387	Methoxyl-isorhamnetin-*O*-glucoside
30^a^	22.35	301.0327	−7.5	C_15_H_10_O_7_	273.0075, 178.9979, 151.0027	Quercetin
31	22.98	343.0821	0.1	C_18_H_16_O_7_	328.0562, 313.0413	Ether-hemerocal
32	23.21	447.0945	2.7	C_21_H_20_O_11_	284.0357	Kaempferol-*O*-glucoside

a Those metabolites were unambiguously identified.

### 2.4 Identification of the metabolites by high-performance liquid chromatography/quadrupole time-of-flight mass spectrometry

**FIGURE 4:**
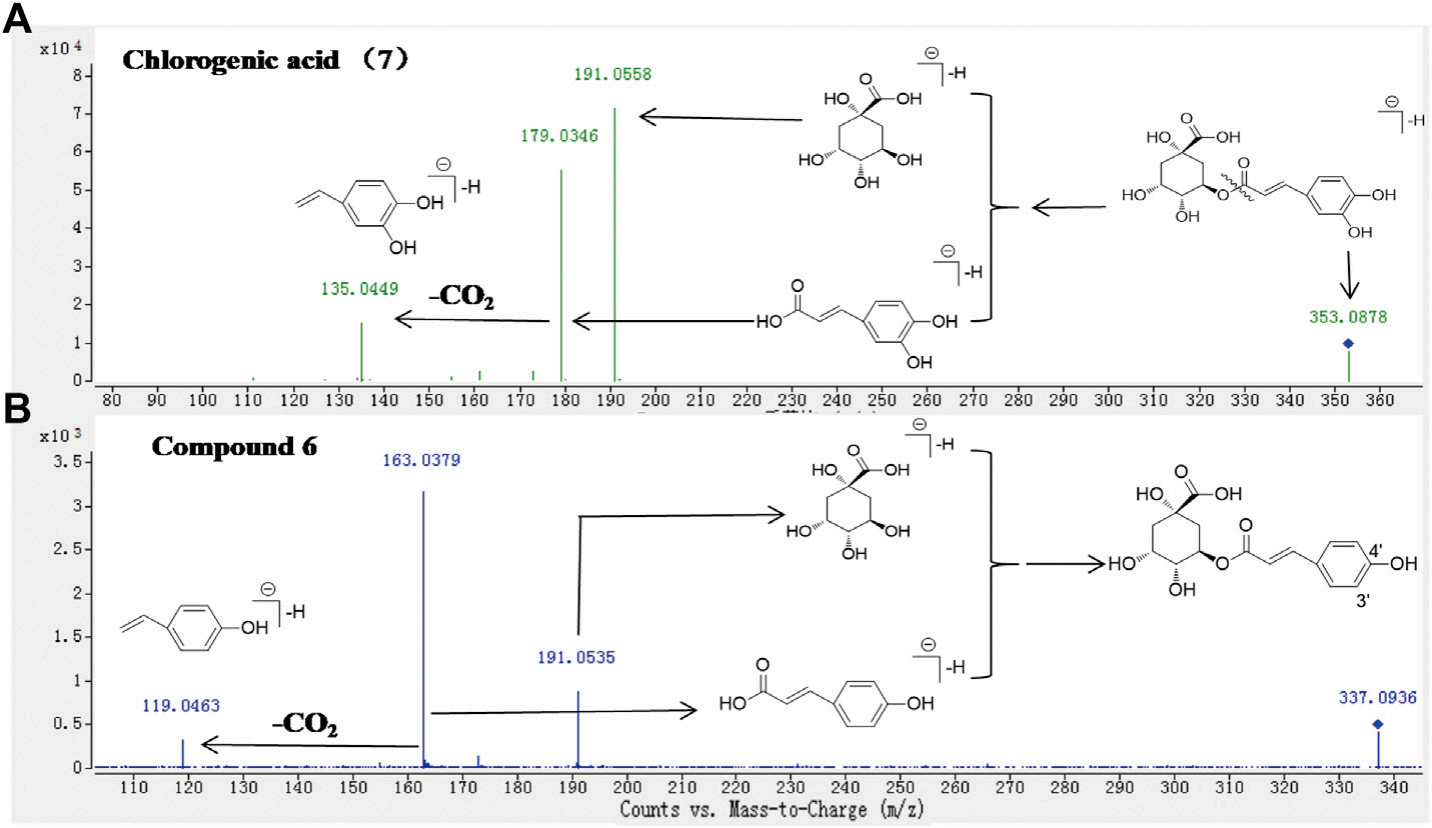
The MS/MS spectra of standard **7 (A)** and the metabolite **6 (B)** in ESI^−^ mode, and corresponding fragmentation behaviors.

HPLC-Q-TOF-MS is a fast and sensitive tool widely used for comprehensive screening and identifying the plant’s metabolite. In this study, each high-level compound which was appeared with a prominent peak in the total ion chromatogram (TIC) or the ultraviolet chromatogram was screened. The MS/MS data of metabolites were obtained by the target-MS/MS methods, and their structures were determined by their characteristic fragmentation behavior. Take metabolites **6** and **7** for example. Compound **7** was unambiguously identified as chlorogenic acid by comparing the retention time, MS and MS/MS data with the standard. The fragmentation pathways of compound **7** were investigated in detail (Figure 4A), and compound **6** has similar fragmentation behaviors to the standard (Figure 4B). The difference *m/z* value of compounds **6** and **7** was 15.9942 Da, which indicated that one of the hydroxyl groups in compound **7** was replaced by a hydrogen atom and formed the structure of **6**. In the MS/MS spectrum of compound **6** (Figure 4B), the high abundance of ions at *m/z* 163.0379 and 119.0463 were formed, which demonstrated that one of the hydroxyl groups connected to the benzene ring was replaced, therefore, compound **6** was tentatively identified as 5-*O*-p-coumaroylquinic acid (Liu et al., 2020). In addition, metabolites **9**, **21**, and **30** were unambiguously identified as caffeic acid, rutin, and quercetin, respectively, by comparing the retention time, MS and MS/MS data with the standards. The rest primary metabolites in the HCW were also identified by their MS/MS spectra (Supplementary Figure S2; Table 1).

### 2.5 Isolation and identification of compound 15

In order to further determine the structure of the primary metabolites, the MS-guided isolation, which was well-developed by our laboratory (Qing et al., 2016; Qing et al., 2017b), was employed. Compound **15** was obtained from HCW and its structure was unambiguously identified by NMR data. Compound **15** was obtained as a yellow amorphous powder. High resolution mass spectrometry (HR-MS) shows molecular ion peak at *m/z* 755.2026 [M-H]^-^, giving a molecular formula of C_27_H_30_O_16_. The ^1^H-NMR and ^13^C NMR data of compounds **15** is as follows: ^1^H-NMR (400 MHz, DMSO), *δ*_H_ 12.66 (1H, s, H-5), 8.36 (1H, s, OH_aglyc._), 7.53 (1H, d, *J =* 8.8 Hz, H-2′), 7.47 (1H, d, *J =* 8.8 Hz, H-6′), 6.83 (1H, d, *J =* 8.4 Hz, H-5′), 6.37(1H, d, H-8), 6.17 (1H, d, H-6), 5.51 (1H, d, *J =* 7.6 Hz, H-1″), 5.05 (1H, d, H-1‴'), 4.38 (1H, d, H-1‴), 3.72(3H, s, 4′-OCH_3_), 4.33, 3.72, 3.48, 3.24, 3.27, 3.16, 3.12(7H, OH), 0.96 (3H, d, *J =* 6.4 Hz, H-6‴), 0.79 (3H, d, *J =* 6.0 Hz, H-6‴'). ^13^C NMR (100 MHz, DMSO), δ 177.66 (C-4), 161.69 (C-5), 157.13 (C-2), 148.79 (C-4′), 133.29 (C-3), 122.49 (C-1′), 118.61 (C-6′), 104.40 (C-10), 101.25 (C-1″), 99.44 (C-6), 94.03 (C-8), 77.62 (C-2″), 76.18 (C-3″), 76.18 (C-4″), 74.34 (C-5″), and 72.67 (C-6″). These data (Supplementary Figure S3) were consistent with the compound named Quercetin3-O-α-L-rhamnopyranosyl-(1→6)-[α-L-rhamnopyranosyl-(1→2)]-β-D-glucopyranoside (Siewek et al., 1984). Compound **15** was reported for the first time from *H. citrina*.

In summary, flavonoids (**14**–**32**) and chlorogenic acid-type compounds (**2**–**4**, **6**–**8**, and **13**) were the primary metabolites of the HCW, which display a crucial role in the anti-depressive activity. Rutin (**21**) was the highest content metabolite of HCW, and compounds **15** and **23** were also the high-level metabolites (Figure 3B), which may have potential anti-depressive activity and need further investigation.

### 2.6 The anti-depressive activity of rutin (compound 21)

**FIGURE 5:**
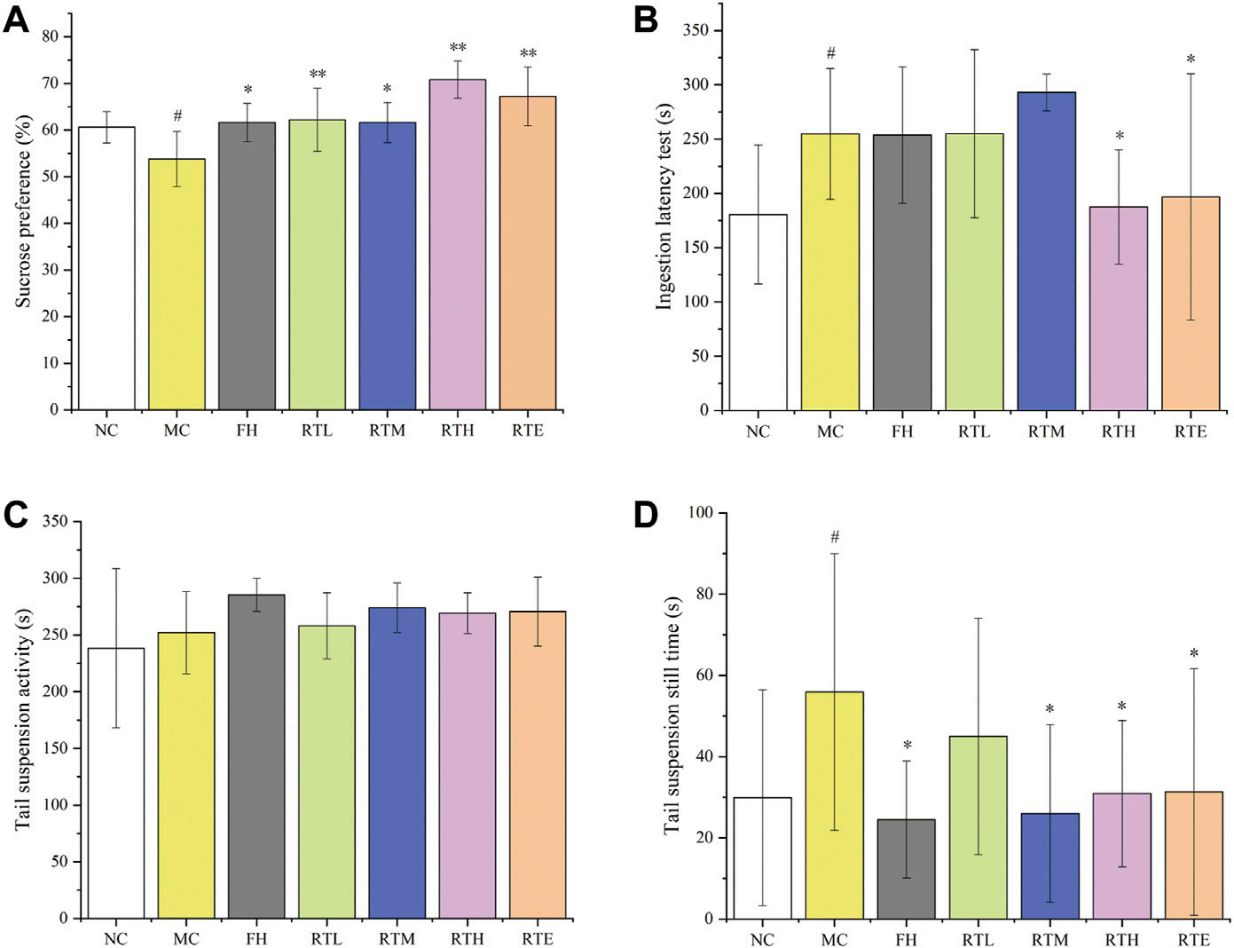
The effects of different dose groups of rutin on the behaviors of CUMS mice. **(A)** Sucrose preference test, **(B)** ingestion latency test, **(C)** tail suspension activity test, and **(D)** tail suspension still time test. Data are reported as mean ± SD. For statistical significant, ^*#*^ *p* < 0.05, ^##^ *p* < 0.01 compared with the normal control group; *p < 0.05, **p < 0.01 compared with the model control group. NC, normal group; MC, model group; FH, Fluoxetine hydrochloride group; RTL, low-dose of rutin (0.7 mg/kg); RTM, medium-dose of rutin (1.8 mg/kg); RTH: high-dose of rutin (6.3 mg/kg); RTE, extra high-dose of rutin (10.0 mg/kg).

In order to find the anti-depressive active component of HCW, the anti-depressive activity of the main metabolite (**21**) was evaluated. The result showed that the SPT index of the model control group was significantly lower (*p* < 0.05) compared with the normal control group, which indicated that the CUMS mice model was successfully established (Figure 5A). Compared to the model group, the SPT index of different doses of rutin (RTL: 0.7 mg/kg, RTM: 1.8 mg/kg, RTH: 6.3 mg/kg and RTE: 10.0 mg/kg, see Supplementary Table S1) and FH group was significantly increased (*p* < 0.05).

As shown in Figure 5B, the ILT of mice in the model control group was significantly prolonged (*p* < 0.05) compared to the normal control group, which also indicated that the CUMS mice model was successfully established. The ILT of mice in the RTH and RHE groups and the FH group decreased significantly (*p* < 0.05) compared to the model control group.

Compared with the normal control group, the resting time of the model control group was significantly prolonged (*p* < 0.05), which showed that the CUMS mice model was successfully established. The resting time of mice in the FH, RTM, RTH, and RTE group was significantly decreased (*p* < 0.05) compared with the model group. However, the tail suspension activity experiment failed (Figures 5C,D).

The results of SPT, ILT, and TST experiments indicated that RTH and RTE have significant anti-depressive activity, which demonstrates that rutin was one of the main anti-depressive active compounds of HCW. And the anti-depressive activities of other high-level compounds such as metabolites **15** and **23** needed further evaluation.

### 2.7 Effect of *H. citrina* flowers on the intestinal flora

Depression is a complicated and comprehensive mood disease, and the pathogenesis is still not completely clear. More and more studies have shown that intestinal flora affects not only gastrointestinal physiology, but also the function and behavior of the central nervous system through the microbiota-intestinal-brain axis (Collins et al., 2012; Rogers et al., 2016). However, the relationships between the antidepressant-like activity of *H. citrina* extracts and intestinal microorganism variations were rarely studied. Therefore, we analyzed the 16S rRNA gene sequencing to determine the effects of two *H. citrina* extracts (low-dose of HCW and HCE) on the gut microbiota of the depressed mice.

### 2.8 *H. citrina* flowers increases the diversity and richness of the intestinal flora

**FIGURE 6:**
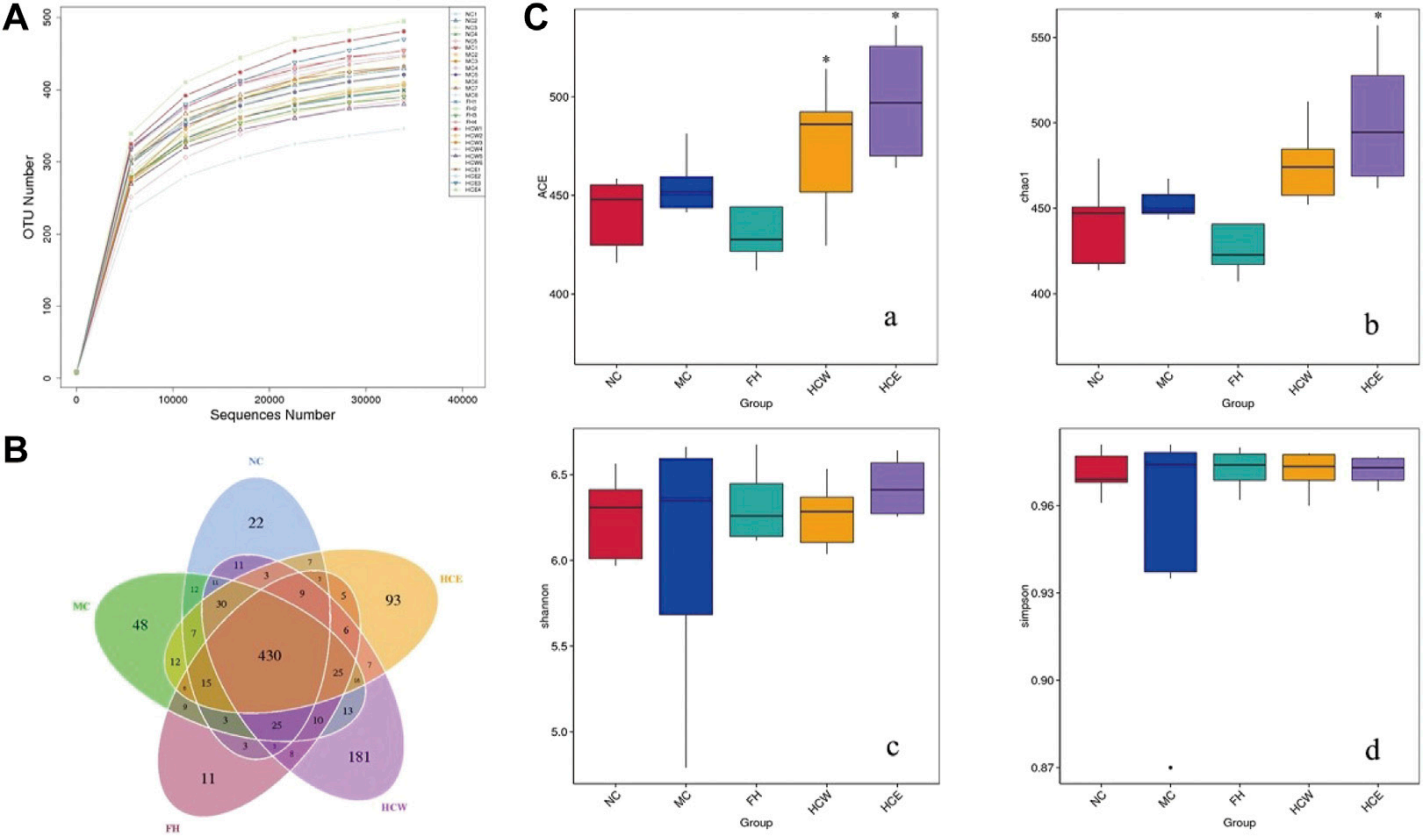
Reflectance curve **(A)** and Venn diagram **(B)** of intestinal microbial OTUs from HCW and HCE-treated CUMS mice and controls, **(C)** the alpha diversity of the ACE, Chao 1, Shannon and Simpson index of intestinal microflora. Data are reported as mean ± SD. For statistical significant, *p < 0.05 compared with the model control group. NC, normal group; MC, model group; FH, Fluoxetine hydrochloride group; HCW, water extract of flowers; HCE, 80% ethanol extract of flowers.

As the depth of sequencing increases, the rarefaction curves of all the samples approach the saturation plateau, and the result indicates that the sequencing data covers all species in the sample (Figure 6A). The Venn diagram compares the differences group between OTUs. A total of 1044 OTUs from five groups of sequencing data of intestinal flora. As shown in Figure 6B, five groups shared 430 OTUs. Unique OTUs were observed in the normal control (22), CUMS model control (48), fluoxetine (11), HCE (93) and HCW (181) groups. *α* diversity included the ACE, Chao1, Shannon and Simpson index, which was intended to be represent the community’s richness and diversity (Liang et al., 2018; Perxachs et al., 2022). As shown in Figure 6C, the HCE and HCW groups exhibited an increase in the alpha diversity of the ACE index compared with the model group (*p* < 0.05), and the HCE group increased in the alpha diversity of the Chao 1 index compared with the model group (*p* < 0.05). The results showed that HCW and HCE treatment improved the diversity and richness of mouse gut microbiota, which was decreased by depression.

Principal coordinates analysis (PCoA) presented gut microbiota communities in mice, which were divided into different quadrants respectively from five groups (Simpson et al., 2021). The model group was separated from the normal group in PCoA space, and the normal group and HCW group exhibited certain polymerization tendencies (Supplementary Figure S4). The result showed that stress stimuli decrease the enrichment and diversity of gut microbiota, and the HCW can reverse that phenomenon.

### 2.9 *H. citrina* flowers regulates the abundance of specific intestinal flora

**FIGURE 7:**
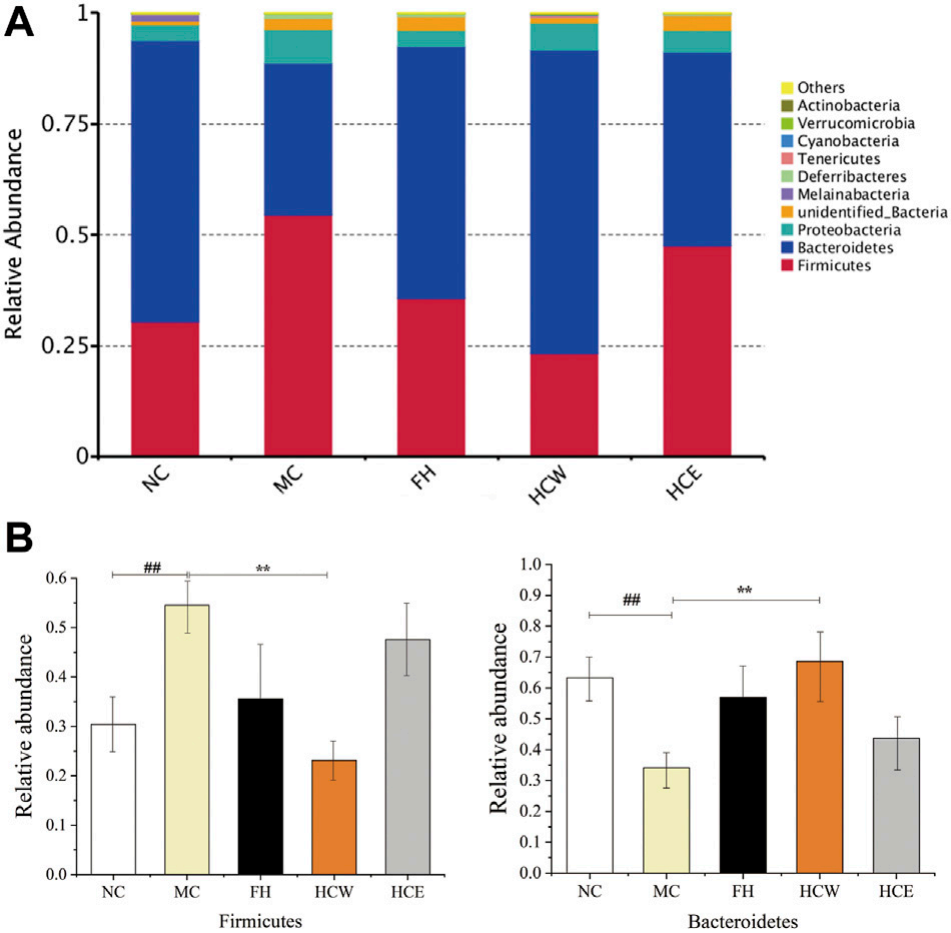
Comparison of the relative abundance of intestinal flora at the phylum level **(A)**. Comparison of *Firmicutes* and *Bacteroidetes* relative abundance in different groups **(B)**. Data are reported as mean ± SD. For statistical significant, ^##^ *p* < 0.01 compared with the normal control group; **p < 0.01 compared with the model control group. NC, normal group; MC, model group; FH, Fluoxetine hydrochloride group; HCW, water extract of flowers; HCE, 80% ethanol extract of flowers.

The effects of CUMS, HCW and HCE on the composition and function of the intestinal flora were analyzed *via* 16S rRNA sequencing. The community composition was analyzed to obtain the abundance and diversity of each species (Pu et al., 2022). At the phylum level, *Firmicutes*, *Bacteroidetes*, and *Proteobacteria* were predominant in all samples but varied in their abundances (Figure 7A). The abundance of *Firmicutes* in the CUMS model group (MC) was significantly higher than that of the normal group (NC) and HCW (*p* < 0.01), which indicated that the HCW could decrease the abundance of *Firmicutes* in the CUMS model group. The abundance of *Bacteroidetes* in the CUMS model group was significantly lower than that of the normal group and HCW (*p* < 0.01), which demonstrated that the HCW could increase the abundance of *Bacteroidetes* in the CUMS model group (Figure 7B). In previous studies, the diversity and abundance of intestinal flora in depressed patients were decreased (Naseribafrouei et al., 2014; Zheng et al., 2016; Lin et al., 2017). Compared to the normal person, at the phylum level, the abundance of *Bacteroidetes* was decreased, and the level of *Firmicutes* was increased in the intestinal flora of depressed patients. In this study, the HCW could increase the abundance of *Bacteroidetes* and decrease the level of *Firmicutes* in the intestinal flora of depressed mice at the phylum level, which indicated that HCW has the potential to be developed as an antidepressant by regulating the abundance of *Firmicutes* and *Bacteroidetes* at the phylum level.

**FIGURE 8:**
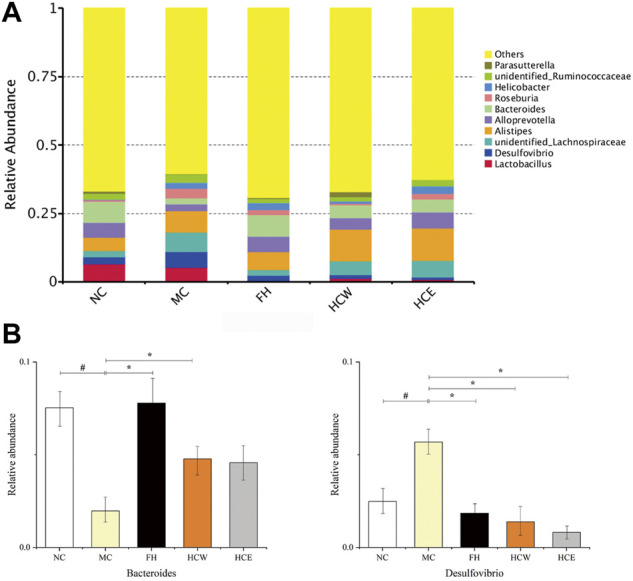
Comparison of the relative abundance of intestinal flora at the genus level **(A)**. Comparison of *Bacteroides* and *Desulfovibrio* relative abundance in different groups **(B)**. Data are reported as mean ± SD. For statistical significant, ^#^ *p* < 0.05 compared with the normal control group; *p < 0.05 compared with the model control group. NC, normal group; MC, model group; FH, Fluoxetine hydrochloride group; HCW, water extract of flowers; HCE, 80% ethanol extract of flowers.

At the genera level, *Bacteroides* and *Desulfovibrio* were the predominant genus in all samples but varied in their abundances (Figure 8A). The abundance of *Bacteroides* was significantly decreased when the normal mice were depressed, while its abundance was significantly increased after administration of the FH and HCW. The abundance of *Desulfovibrio* were significantly increased when the normal mice depressed, while their abundance was significantly decreased after administration of the HCW (Figure 8B). The above results indicated that the HCW regulates the abundance of *Bacteroides* and *Desulfovibrio* at genera level to achieve the anti-depressive activity based on the microbiota-intestinal-brain axis.

Gamma-amino butyric acid (GABA), which is an important neurotransmitter, was used to transmit signals in the synapses of the nervous system. In previous studies (Ironside et al., 2021; Prévot and Sibille, 2021), the level of GABA significantly decreased in depressive patients, including major depressive disorder (MDD) patients. Previous studies indicated that the intestinal flora belonging to *Bacteroides* genera could produce GABA in the gut, which has an effect on the synapses of the nervous in the brain by the microbiota-intestinal-brain axis (Strandwitz et al., 2019; Izuno et al., 2021; Otaru et al., 2021). In this study, the HCW could significantly increase the abundance of *Bacteroides* genera in the gut of depressed mice, which indicated that the HCW plays the anti-depressive activity by increasing the abundance of *Bacteroides* genera and thereby improving the level of GABA.

*Desulfovibrio* genera could produce the lipopolysaccharide to disrupt the intestinal barrier and leads to the production of inflammatory factors. In previous studies (Haroon and Miller, 2017; Zhu et al., 2019), the abundance of *Desulfovibrio* genera was significantly increased in the intestinal flora of depressed mice, which was in accordance with the results of this study. The inflammatory factors, such as IL-1β, IL-6 and TNF-α, could damage the epithelial cells of the gut and have an effect on the frontal cortex and hippocampus of the brain by the blood circulation, which could lead to depression (Chudzik et al., 2021; Morais et al., 2021). In this study, HCW could significantly decrease the level of *Desulfovibrio* genera of depressive mice. The potential mechanism involved in decreasing the level of lipopolysaccharide produced by *Desulfovibrio* genera and thereby reducing the content of inflammatory factors in the gut, blood, and brain of depressed mice.

### 2.10 Effect of rutin on the intestinal flora

Rutin (**21**) is a polyphenolic compound that has been proven to have antidepressant activity. However, the relationship between the antidepressant-like activity of rutin and intestinal microorganism variations was rarely reported. Therefore, we analyzed the 16S rRNA gene sequencing to investigate the effects of rutin (high-dose RT, namely RTE) on the gut microbiota of depressed mice.

### 2.11 Rutin increases the diversity and richness of the intestinal flora

According to the sample number and species OUTs, the rarefaction curves of all the samples had reached a plateau, which indicated that the sequencing data covers all species in the sample (Supplementary Figure S5A). A total of 1205 OTUs from four groups of sequencing data of intestinal flora. As shown in Supplementary Figure S5B, four groups shared 792 OTUs. Unique OTUs were observed in the normal control (35), CUMS model control (13), FH (21), and RT (33) groups. As shown in Supplementary Figure S5C, the model group was separated from the normal group in PCoA space, and the normal group and RT group exhibited certain polymerization tendencies. The FH and RT groups exhibited an increase in the alpha diversity of the ACE index, Chao 1 index, and Shannon index compared with the model group (*p* < 0.05), and the model group decreased the alpha diversity of the ACE index and Chao 1 index compared with the normal group (*p* < 0.05) (Supplementary Figures S5A–D). The results showed that FH and RT could improve the diversity and richness of the gut microbiota of depressed mice.

### 2.12 Rutin regulates the abundance of specific intestinal flora

At the phylum level, *Firmicutes*, *Bacteroidetes*, and *Proteobacteria* were predominant in all samples but varied in their abundances (Supplementary Figure S7A). Although the variation trend of the abundance of those microbiotas was consistent with the HCW group. However, the RT group did not display significant function in regulating *Firmicutes* and *Bacteroidetes* phylum levels in the depressed mice (*p* > 0.05). At the genera level, the RT group could increase the abundance of *Bacteroides* and decrease the level of *Desulfovibrio* genera (Supplementary Figure S7B), which was in accordance with the HCW group. However, the effect of the RT group on the level of *Bacteroides* and *Desulfovibrio* genera in depressed mice was weaker than the HCW group. The result shows that rutin is one of the main ingredients of anti-depression in HCW.

## 3 Materials and methods

### 3.1 Preparations of *H. citrina* extracts

Dry, fresh flower buds and flowers of *H. citrina* (Mengzihua, 100 Kg each, Supplementary Figure S1) were collected from Qidong County, Hunan Province of China, and were unambiguously identified by Doctor Zhixing Qing (Hunan Agricultural University). The extract experiments used water and 80% ethanol as the extraction solvent. The ratio of material to liquid was 6: 1 and the extraction time was 24 h under room temperature. The extraction solvents were concentrated by reducing pressure and dried by vacuum. Finally, 6 different extracts, which include water extracts of flowers (HCW), water extracts of fresh flower buds (WHCW), 80% ethanol extracts of flowers (HCE), 80% ethanol extracts of fresh flower buds (WHCE), water extract of dried flower buds (DHCW), and the 80% ethanol extract of dried flower buds (DHCE), were obtained for the anti-depressive experiments.

### 3.2 Chemicals

Acetonitrile and formic acid (HPLC-grade) were purchased from Merck (Darmstadt, Germany) and ROE (Newark, New Castle, United States), respectively. Deionized water was purified using a Milli-Q system (MA, United States). All of them were used for HPLC-Q-TOF-MS analysis. Three standards, including chlorogenic acid (**7**), rutin (**21**) and quercetin (**30**), were purchased from Chengdu Must Bio-Technology Co., Ltd. (Sichuan, China). Fluoxetine hydrochloride tablets were used as positive control drugs (batch number: 16090487345A, Products of Lilai Suzhou Co., Ltd.). Biochemical reagents, including E.Z.N.A.^®^, Soil DNA Kit (Omega Bio-Tek, United States), FastPfu Polymerase (TransGen, China), AxyPrep DNA Gel Extraction Kit (Axygen, United States), NEXTFLEX^®^, Rapid DNA-Seq Kit (Bioo Scientific, United States), MiSeq Reagent Kit v3 (Illumina, United States) and agArose (Biowest, Spain) were purchased from Solarbio Science and Technology Co., Ltd. (Beijing, China) and used for 16S rRNA analysis.

### 3.3 High-performance liquid chromatography/quadrupole time-of-flight mass spectrometry conditions

Chromatography was performed using an Agilent 1290 HPLC system (Agilent Technologies, United States) consisting of an auto-sampler, thermostatted column compartment, and a tunable UV detector. Separation was carried out on a XAqua C18 (150 mm × 2.1 mm, 2.8 µm; Accrom Technologies Co. Ltd., China). The elution system was 0.1% formic acid 1) and 0.1% formic acid in acetonitrile 2). The linear gradient elution program was as flowers: 0–30 min, 5%–45% B; 30–40 min 45%–90% B. The sample injection volume was 5 μl. The rate was set at 0.3 ml/min, and the column temperature was maintained at 30°C.

Mass spectrometric experiments were performed using a 6530 Q-TOF/MS accurate mass spectrometer (Agilent Technologies, United States) in negative ionization mode, and TOF data were acquired between *m/z* 100 and 1000 in centroid mode. The condition of Q-TOF-MS was optimized as follows: sheath gas temperature: 350°C; sheath gas flow, 12 L/min; gas temperature, 300°C; drying gas, 10 L/min; fragmentor voltage, 150 V; skimmer voltage, 65 V, capillary voltage, 4000 V. The TOF mass spectrometer was continuously calibrated using a reference solution (masses at *m/z* 112.9855 and 966.0007) to obtain the high-accuracy mass measurement. The targeted MS/MS experiments were performed using variable collision energy (10–50 eV), which was optimized for each metabolite.

### 3.4 Mass-guided isolation of the high-level metabolite 15

Approximately 50 g extract was subjected to column chromatography (CC) on the macroporous resin (AB-8, 1 kg, 50 cm× 10 cm) with an EtOH-H_2_O gradient (0:100, 30:70, 50:50, and 100:0) to yield four fractions (Fr.1–Fr.4). Fr.1 to Fr.4 were tested by HPLC-Q-TOF-MS and metabolite **15** was detected from Fr.2 (Supplementary Figure S8, R_*t*_ = 11.2 min), which was further separated by preparative HPLC (Agilent ZORBA SB-C18 (4.6 mm × 250 mm, 5 um), HCN/0.1% formic acid, 30:70, 1.0 ml/min, 254 nm) to yield compound **15** (20.2 mg).

### 3.5 Experimental animal

Male ICR mice (weight range of 18.0–22.0 g) were purchased from Hunan Slake Jing-da Experimental Animals Co., Ltd. (Certificate number 43004700048590). The experimental animal production license number is SCXK (Xiang) 2011-0003, and the use license number is SYXK 2015-06. Animals were housed under a standard 12: 12 h light/dark schedule with the light on at 8:00 a.m. and given free assess to tap water and food pellets. The ambient temperature was controlled at (22°C ± 2°C) and given a standard chow and water *ad libitum* for the duration of the study. All experiments and procedures were carried out according to the Regulations of Experimental Animal Administration issued by the State Committee of Science and Technology of China.

### 3.6 Establishment of depression model by chronic unpredictable mild stress

In addition to 10 mice in the normal control group, the other mice were subjected to a chronic unpredictable mild stress, including fasting (12 h), water prohibition (12 h), forced swimming (10 min), strobe (12 h), noise (30 min), restraint (12 h) (placed in a 50 ml centrifuge tube with a diameter of 3.0 cm, a length of about 10 cm, and 6 to 7 vents with a diameter of 0.5 mm), tilting cage (12 h), wet cage (12 h), reversal of day and night, etc. (specific schedule see Supplementary Table S2). Animals abstain from food and water during bondage (Lu et al., 2019).

### 3.7 Drug administration

After 28 days of continuous modeling, the animals were randomly divided into several groups (Supplementary Tables S1, S3, S4, three anti-depressive trails have been done in this study) according to the results of the sucrose preference test and body weight. The normal and model control groups were given distilled water by intragastric administration, and the other groups were given the corresponding solution of extracts with a volume of 20 ml/kg for continuous 35 days. After the last administration, the mice were tested for SPT, ILT, and TST. All antidepressant experimental procedures are shown in Supplementary Figure S9.

### 3.8 Behavior tests

#### 3.8.1 Sucrose preference test

The sucrose preference test was divided into training and test period. Two days before the test as the training period, two bottles of 1% sucrose solution were given to the animals in the first 24 h, and a bottle of 1% sucrose solution was replaced with a bottle of pure water in the next 24 h. They did not abstain from food and water for 8 h before the test. During the test period (15 h), mice were given a bottle of 1% sucrose solution and a bottle of pure water, and the positions of the two bottles were swapped to avoid the influence of position preference. At the end of the test, calculate the sucrose preference index (sucrose preference index (%) = sucrose consumption/(sucrose consumption + pure water consumption) × 100%).

#### 3.8.2 Ingestion latency test

The ingestion latency test was carried out after the SPT. The experiment was divided into 2 days, the first day was the adaptation period, and the animals were put into a square open box to adapt for 10 min. After fasting for 24 h, the ingestion latency test was carried out. A food pellet was placed in the center of the open box, and the mice were put back to assess the food pellet (mice were placed in the same position and direction each time). The time between the animal was put into the cage and the first ingestion of food was recorded.

#### 3.8.3 Tail suspension test

After the ILT, the mice were tested by tail suspension test. The mice were fixed on the tail suspension device with a baffle to separate the line of sight and avoid interfering with others mice. The head was about 5 cm from the table, so the mice had no place to climb onto or grasp. The activity and rest time of the mice were recorded in the next 4 min.

#### 3.8.4 Collection of intestinal solutes

All mice were anesthetized in a container filled with ether gas after the behavioral test, and decapitated quickly to avoid more pain. The intestines of the experimental mice were cut off under the aseptic environment, and the intestinal solutes were dug up with the aseptic knife. The intestinal solutes were collected with sterilized centrifuge tubes on the ice bag and stored in the refrigerator at −80°C for 16S rRNA analysis.

#### 3.8.5 DNA extraction and PCR amplification

The DNA of mice intestinal samples was extracted according to the instructions of “E.Z.N.A.^®^ Soil DNA Kit”. Each sample of DNA was diluted to 1 ng/µl with sterile water, and measured the purity and concentration *via* agarose gel electrophoresis. PCR was performed using TransGen AP221-02, and a specific primer (16S V4 region primers, 515F and 806R) with barcode and amplification was used with related enzymes. 1% agarose gel electrophoresis was used to detect PCR amplification product and the target band was recovered by shearing. Take the qualified PCR amplification product and send it to Shanghai Meiji Biomedical Technology Co., Ltd. for sequencing.

#### 3.8.6 Library construction and computer sequencing

The library was constructed using NEXTFLEX Rapid DNA-Seq Kit. The V4 region of the 16S rRNA gene was analyzed by high throughput sequencing using the Illumina HiSeq platform by NovaSeq PE250. After sequencing is completed, the data are subjected to low-quality read removal, splicing, filtering, and chimera removal to obtain valid data. Sequences were analyzed using Quantitative Insights into Microbial Ecology software and the UPARSE pipeline.

#### 3.8.7 Statistical method

SPSS16.0 was used for experimental data statistical analysis, and the statistically significant level was set to *p* ≤ 0.05. The data were expressed as mean ± standard deviation. Leven’s test method was used to test normality and homogeneity of variance. Multiple samples were compared through one-way ANOVA, and the LSD test was used for statistical analysis. Statistical differences and biological significance were considered in the evaluation.

## 4 Conclusion

In this study, the anti-depressive activities of six extracts of *H. citrina* (HCW, HCE, WHCW, WHCE, DHCW, and DHCE) were evaluated by the depressive mice induced by CUMS model. The results showed that the extracts of *H. citrina* flowers (HCW and HCE) displays significant anti-depressive activities and the HCW has the strongest function than other extracts. A total of 32 compounds, which were mainly flavonoids and chlorogenic acid-type compounds, were identified by HPLC-Q-TOF-MS/MS and NMR. Among them, the content of rutin (compound **21**) was the highest. And then, the anti-depressive activities of rutin was also estimated, the results showed that this compound displayed significant anti-depressive activity and was one of the main active compounds of HCW. Finally, the 16 s (V3+V4) region amplifiers of mice intestinal flora were sequenced to explore the mechanisms of HCW and rutin on intestinal microflora. The results indicated that HCW and rutin could increase the diversity and richness of the intestinal flora, and regulate the specific intestinal microorganisms of the depressed mice. To sum up, the water extract of *H. citrina* flowers (HCW) has significant antidepressant activity, and its main active metabolites were determined and the related mechanism has been proposed.

## Data Availability

The original contributions presented in the study are included in the article/Supplementary Material, further inquiries can be directed to the corresponding authors.
