# Supplementary material for: Antidepressant-like activity, active components and related mechanism of Hemerocallis citrina Baroni extracts
Source: Front Pharmacol. 2022 Aug 29;13:967670. doi: 10.3389/fphar.2022.967670 (PMC9469015; doi:10.3389/fphar.2022.967670)
Supplement: Supplementary file 1 [file DataSheet1.doc]

**Antidepressant-like activity, active metabolites and related mechanism of *Hemerocallis citrina* Baroni extracts**

Jinghong Liu^1,3,†^, Tian Ye^2,†^, Shuaiyong Yang^2^, Xiaohong Zhong^1^, Wei He^4^, Mengtao Xu^2^, Jinpeng Fang^2^, Miao Deng^6^, Ning Xu^1^, Jianguo Zeng^2,5*^, and Zhixing Qing^2,5*^

^1^College of Horticulture, Hunan Agricultural University, Changsha, 410128, China

^2^Hunan Key Laboratory of Traditional Chinese Veterinary Medicine, Hunan Agricultural University, Changsha, 410128, China

^3^Department of Bioengineering and Environmental Science, Changsha University, Changsha, 410003, Hunan, China

^4^Green Melody Bio-engineering Group Company Limited, Changsha 410329, China

^5^Datong Daylily Industial Development Research Institute, Shanxi, Datong, China

^5^College of Food Science and Technology, Hunan Agricultural University, Changsha, 410128, China

*Correspondence should be addressed to Dr. Zhixing Qing and Prof. Jianguo Zeng at the following address: Hunan Key Laboratory of Traditional Chinese Veterinary Medicine, Changsha, 410128, China. Tel.: +86-731-84686560; Fax: +86-731-84686560. E-mail address: qingzhixing@hunau.edu.cn (Zhixing Qing); [zengjianguo@hunau.](mailto:zengjianguo@hunau.net)edu.cn (Jianguo Zeng).

† These authors contributed equally to this work.

Fig. S1 The dried (A), fresh flower buds(B) and flowers (C) of *H. citrina*. (The fresh flower buds of *H. citrina* were washed in 100 ℃ water for 60 seconds and then put into the electric blast drying equipment at 70 ℃ until the moisture content was less than 15%. Finally, the dried flower buds were obtained and employed for anti-depression experiments)


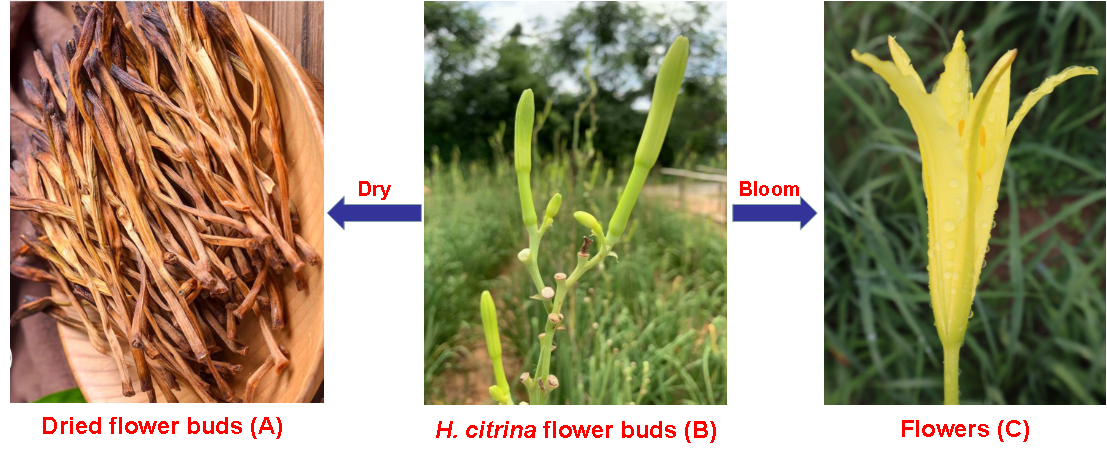


Fig. S2 the MS/MS spectra of identified metabolites **1**-**32**.


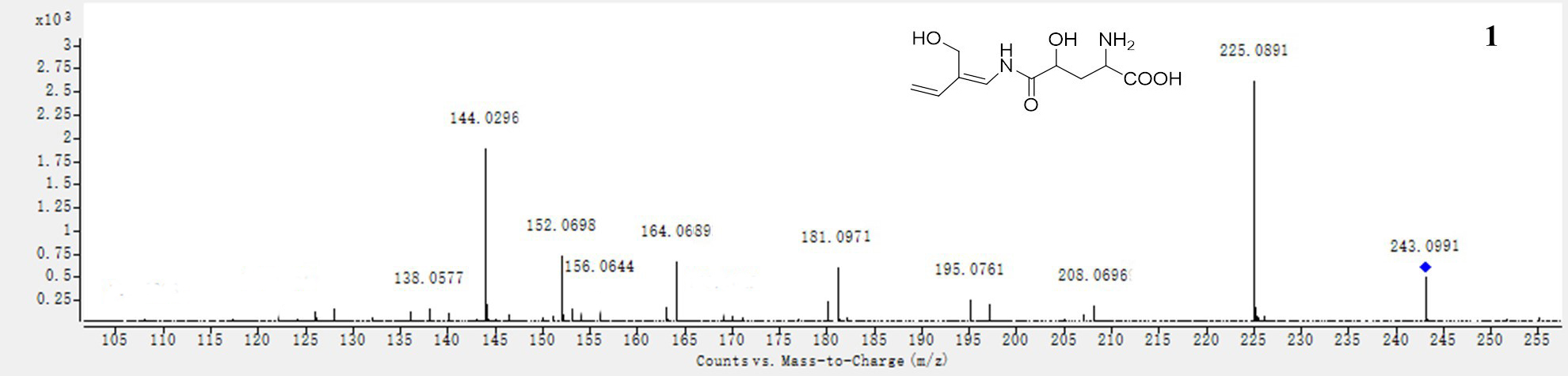


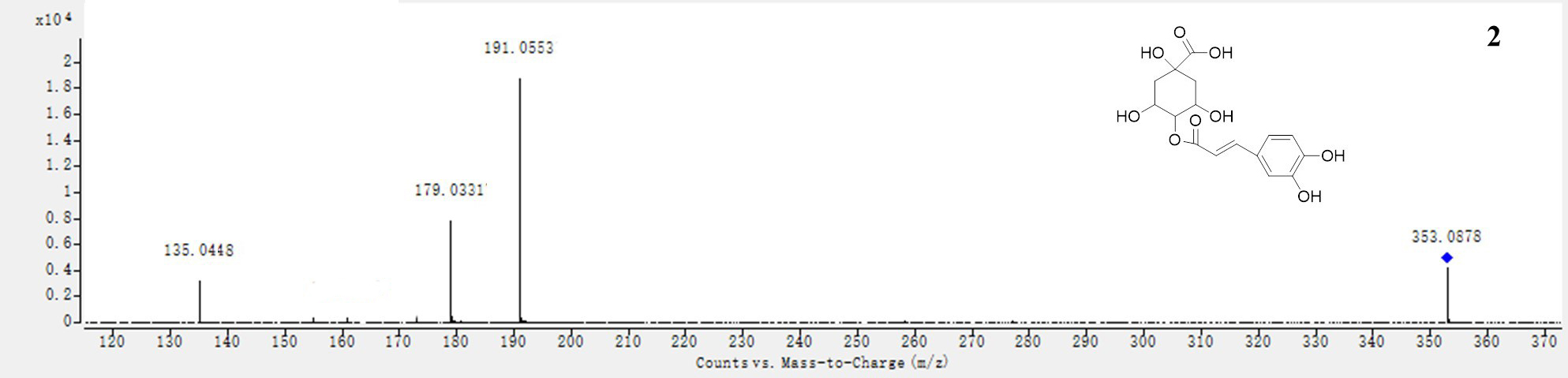


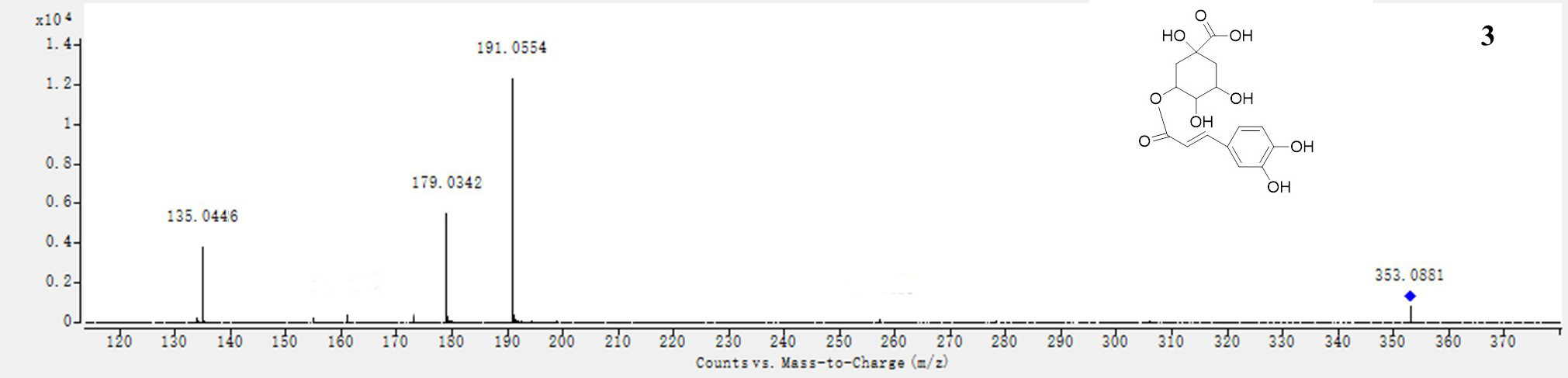


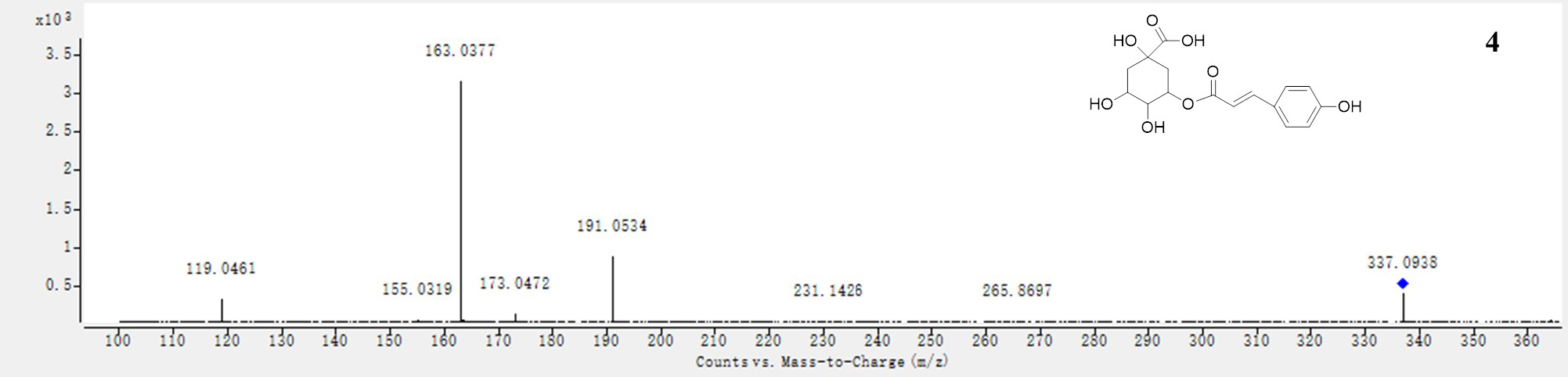


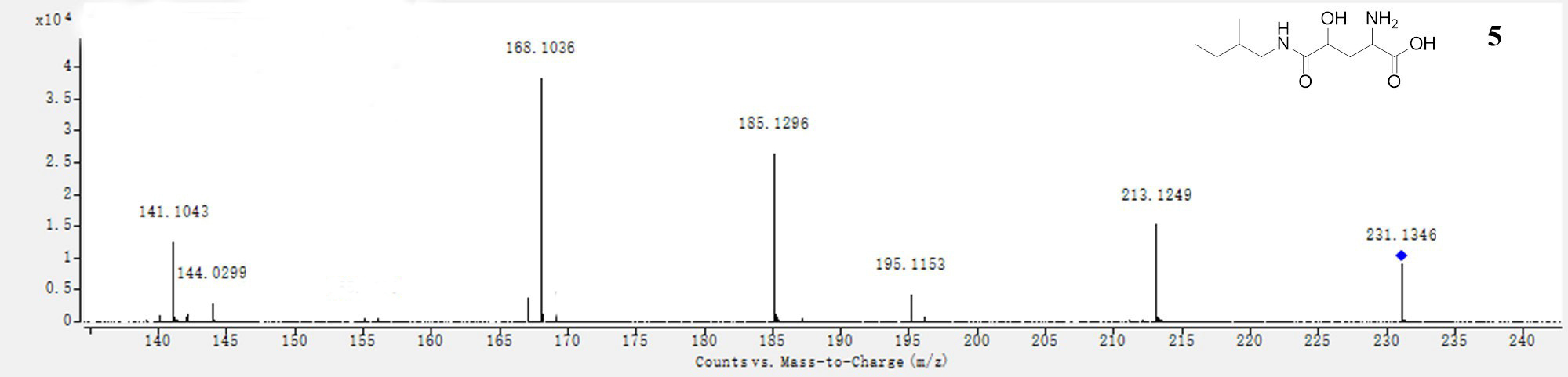


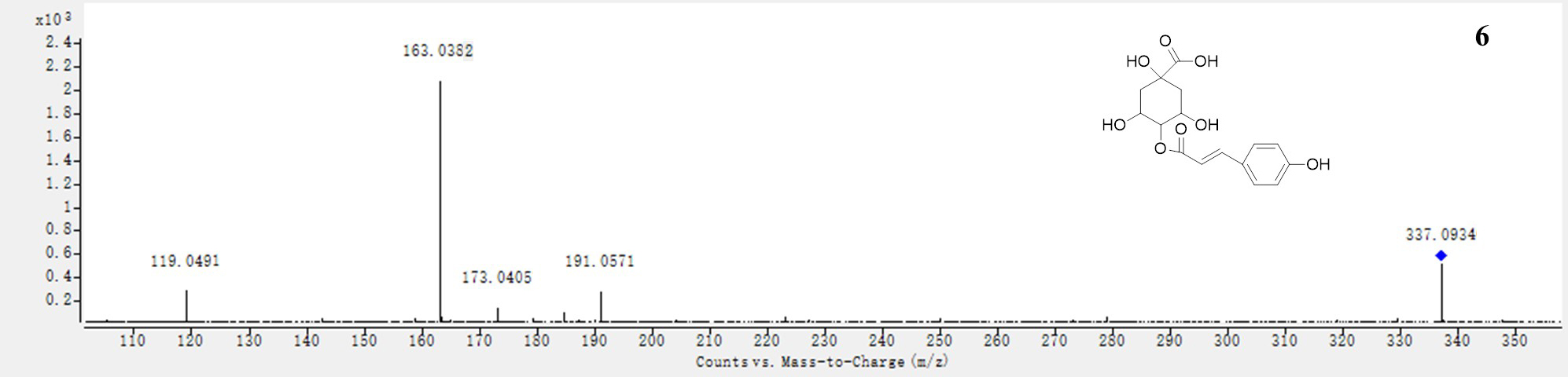


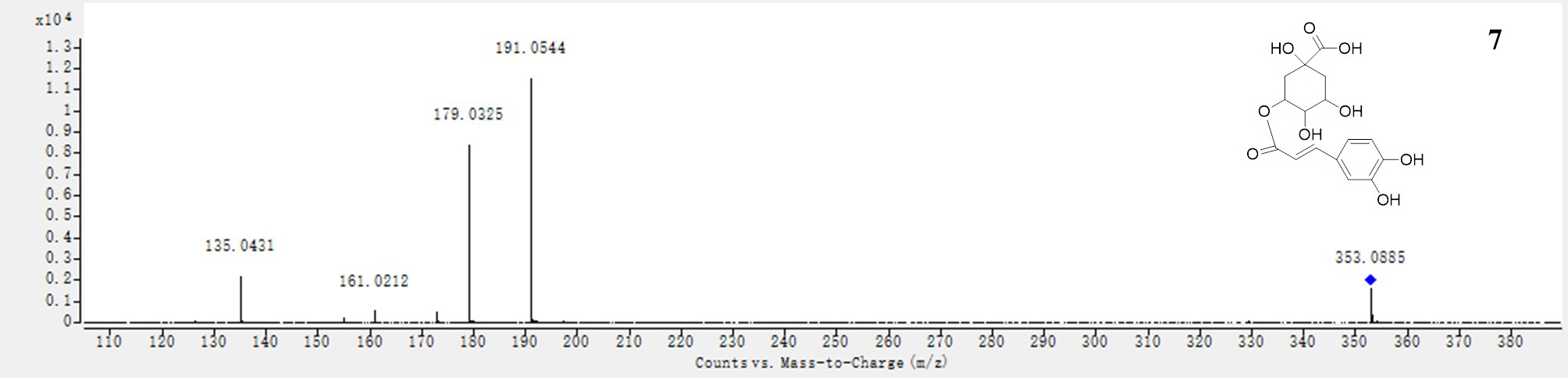


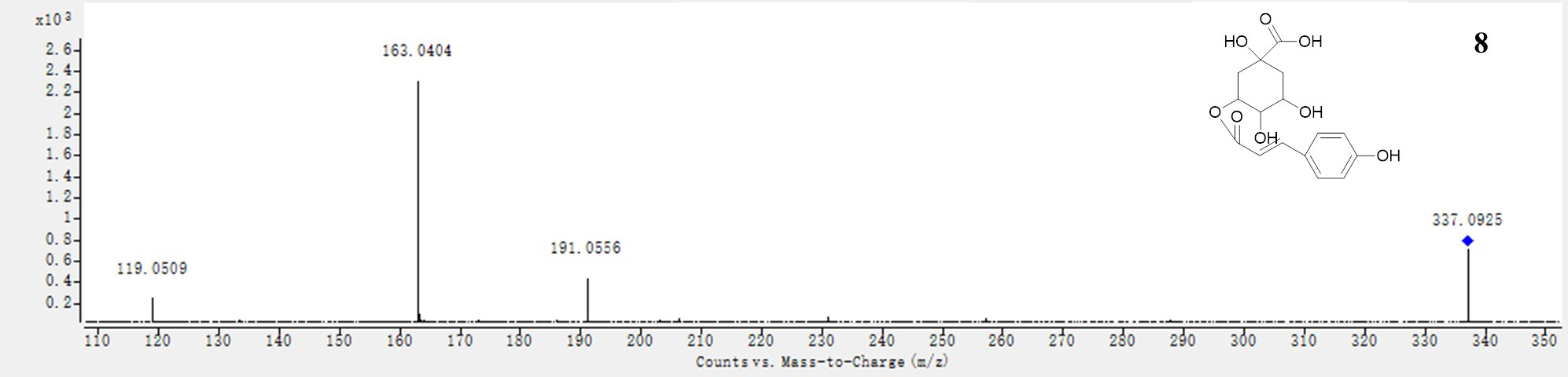


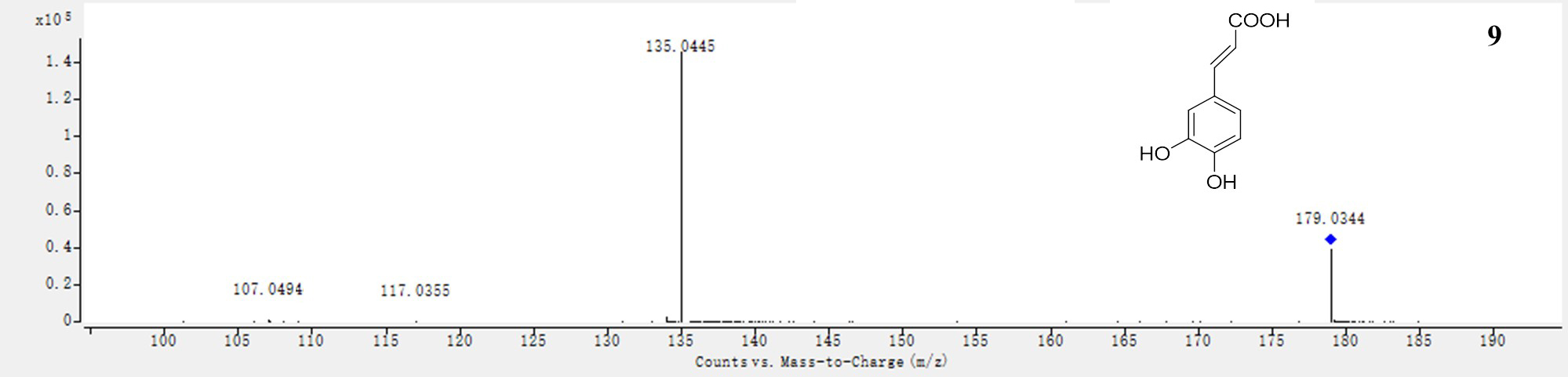


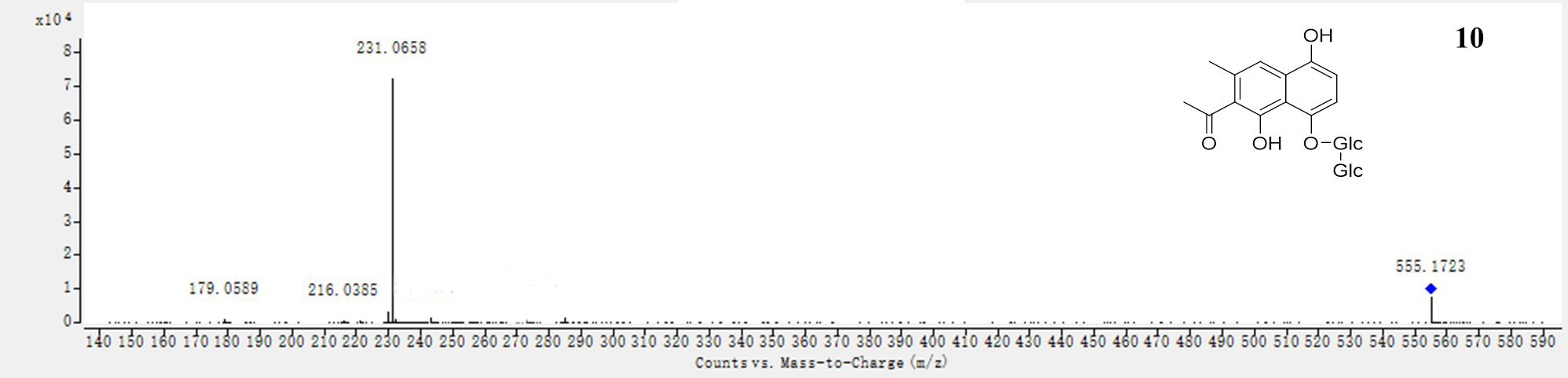


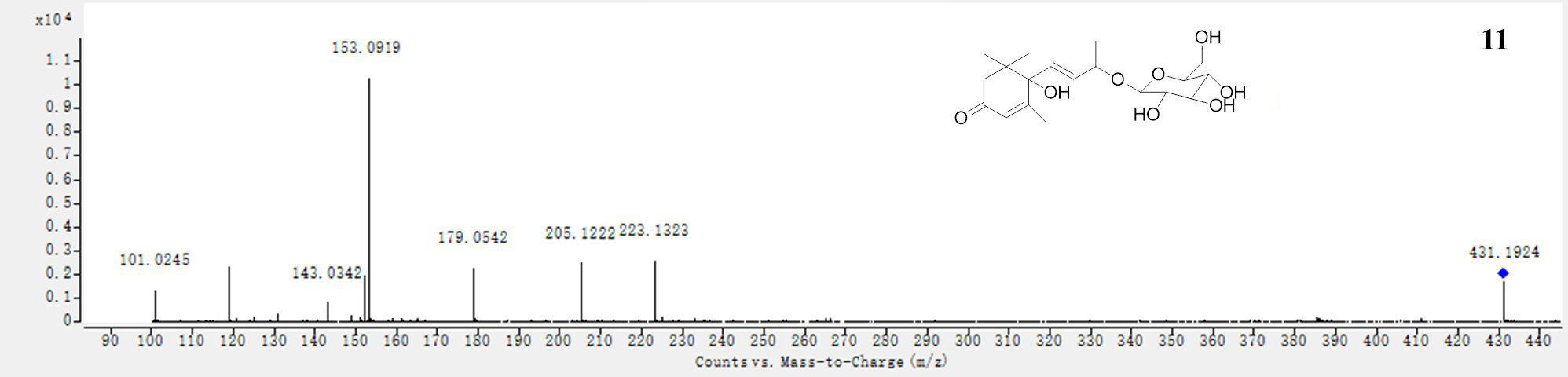


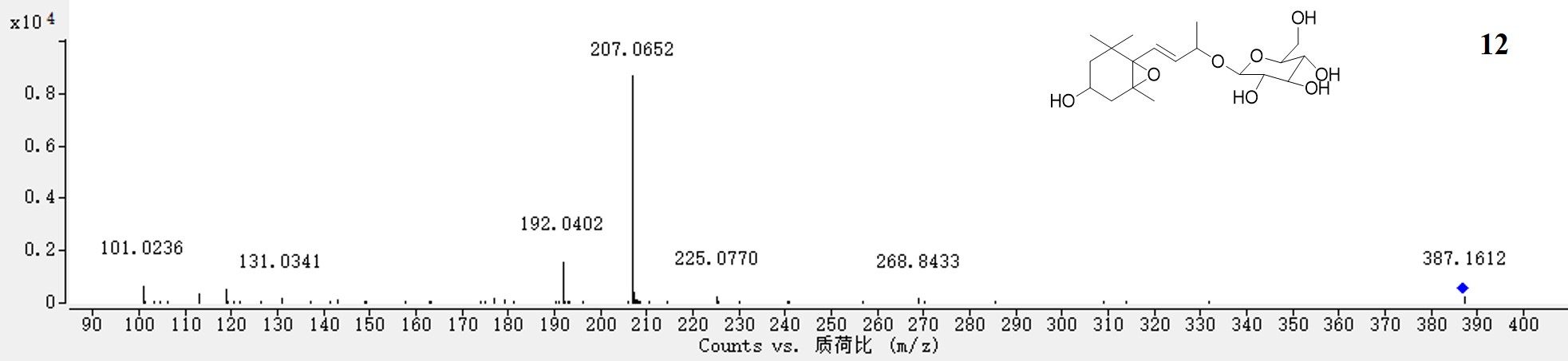


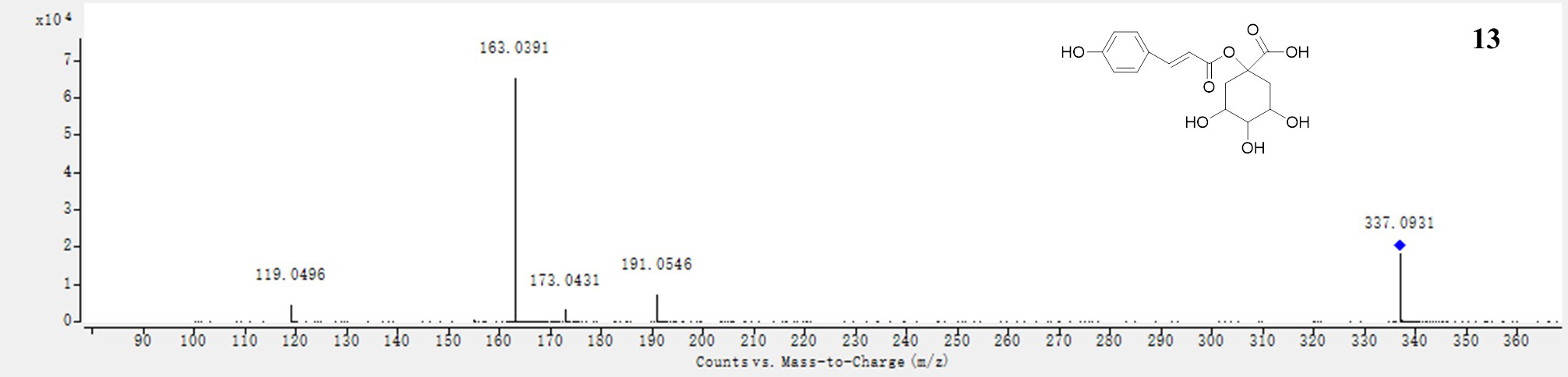


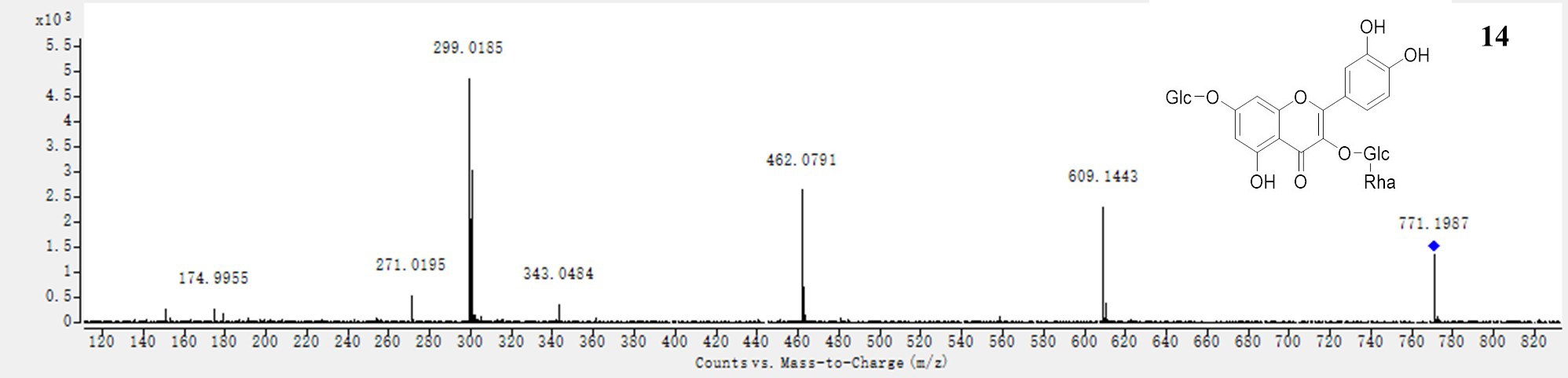


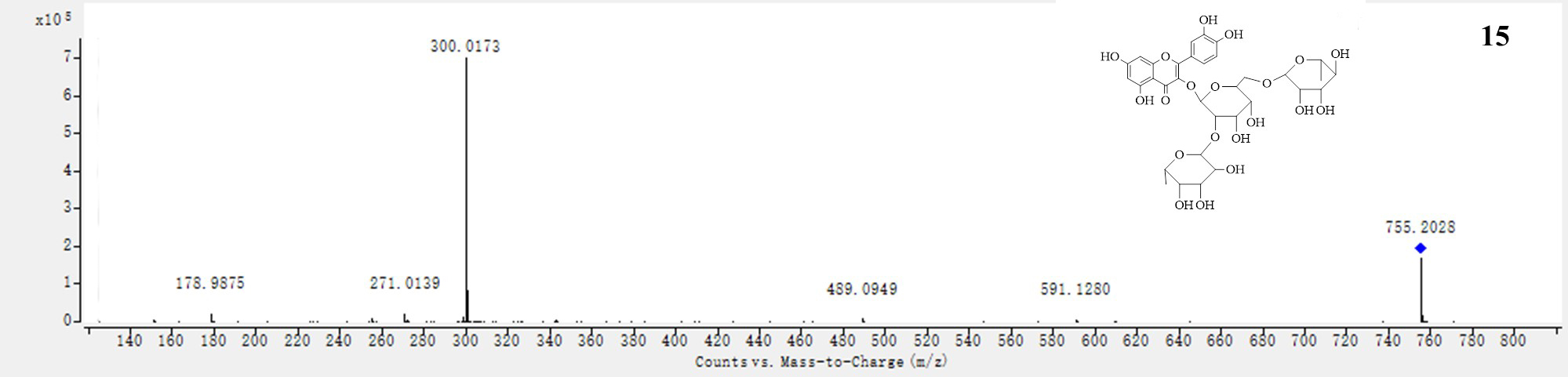


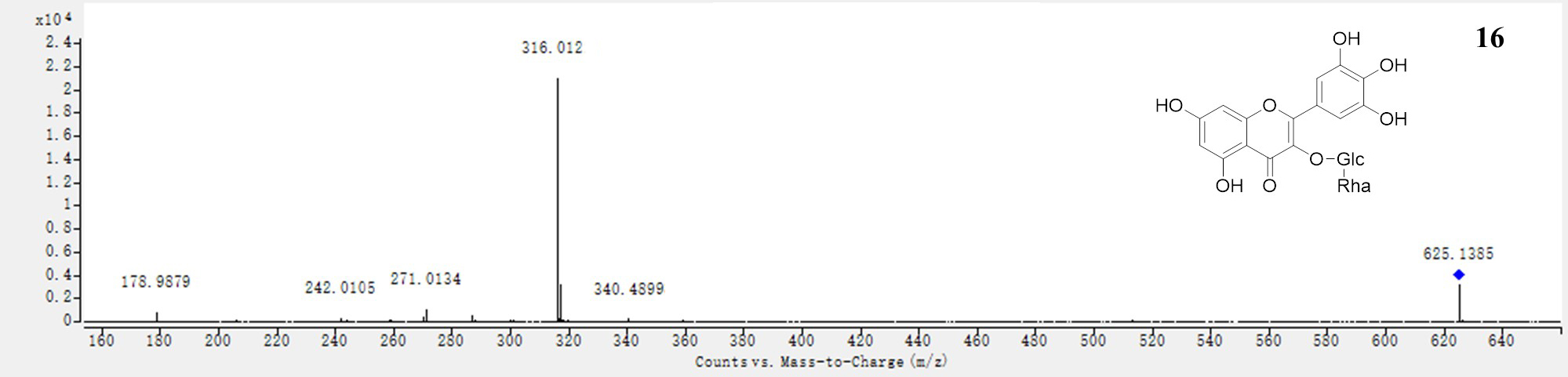


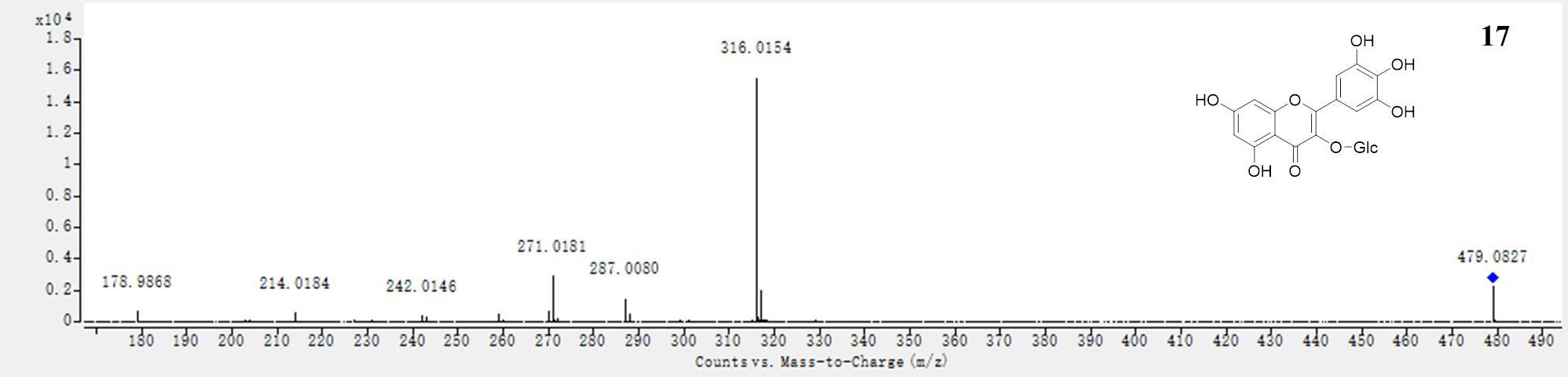


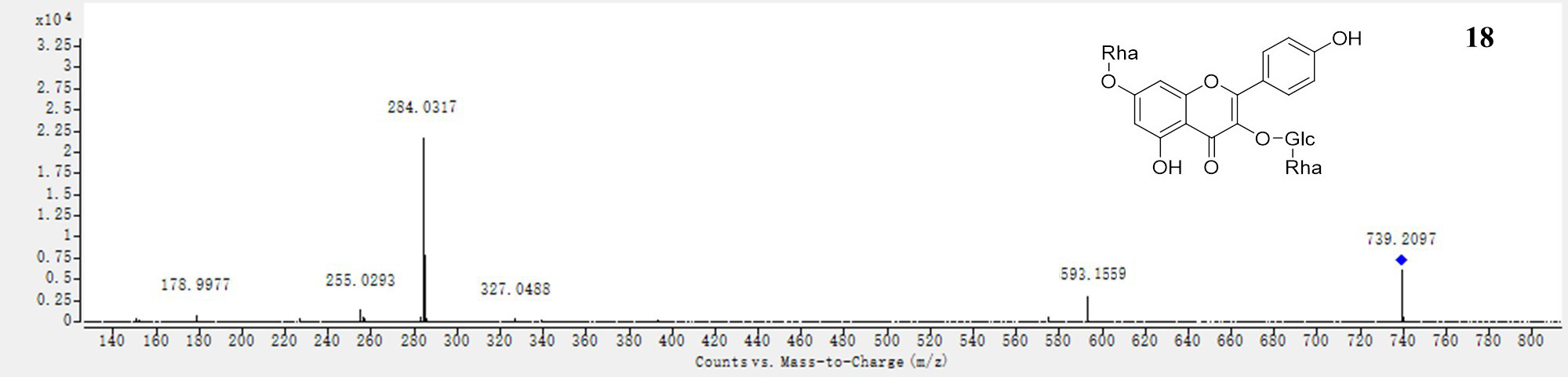


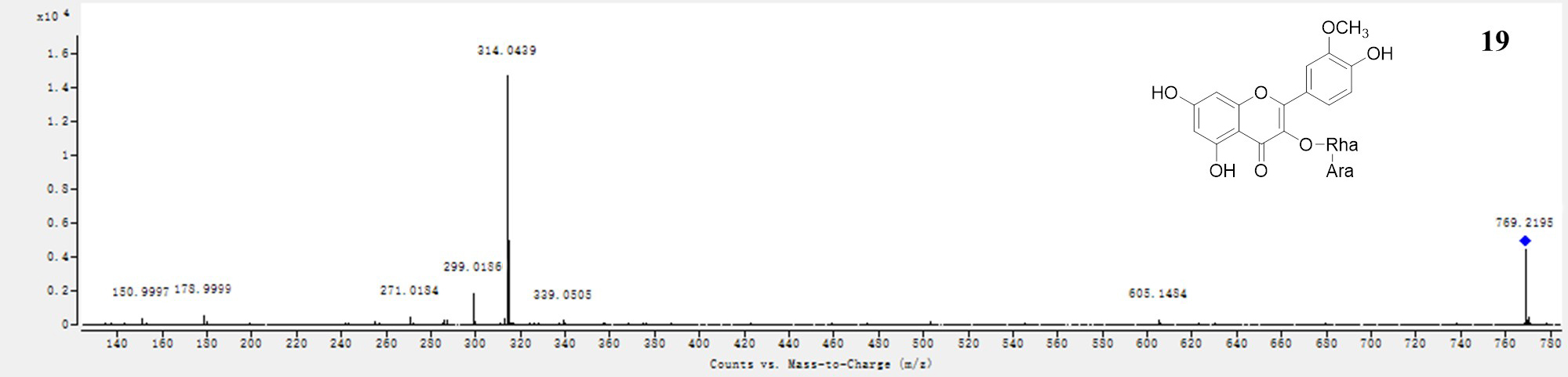


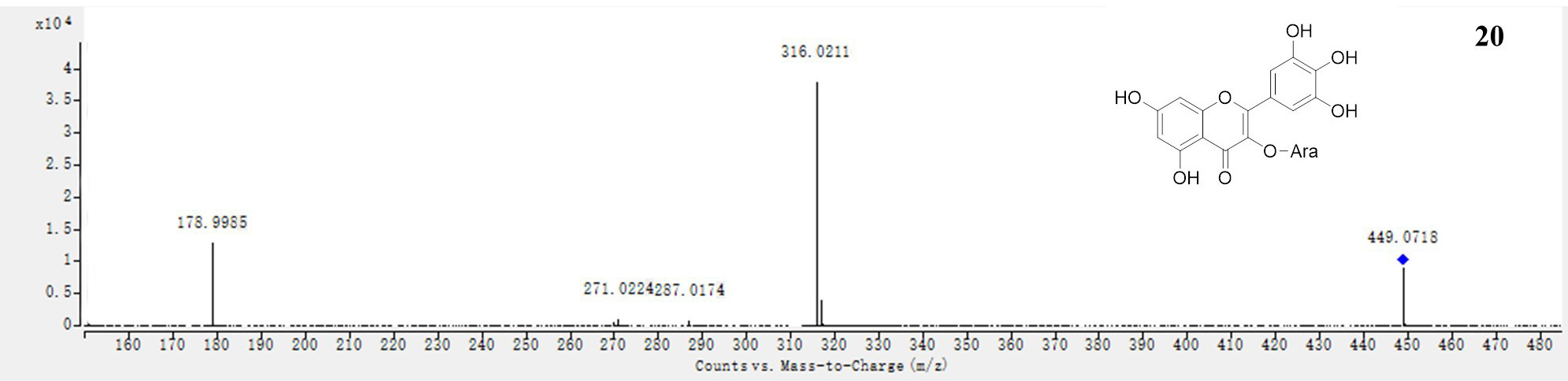


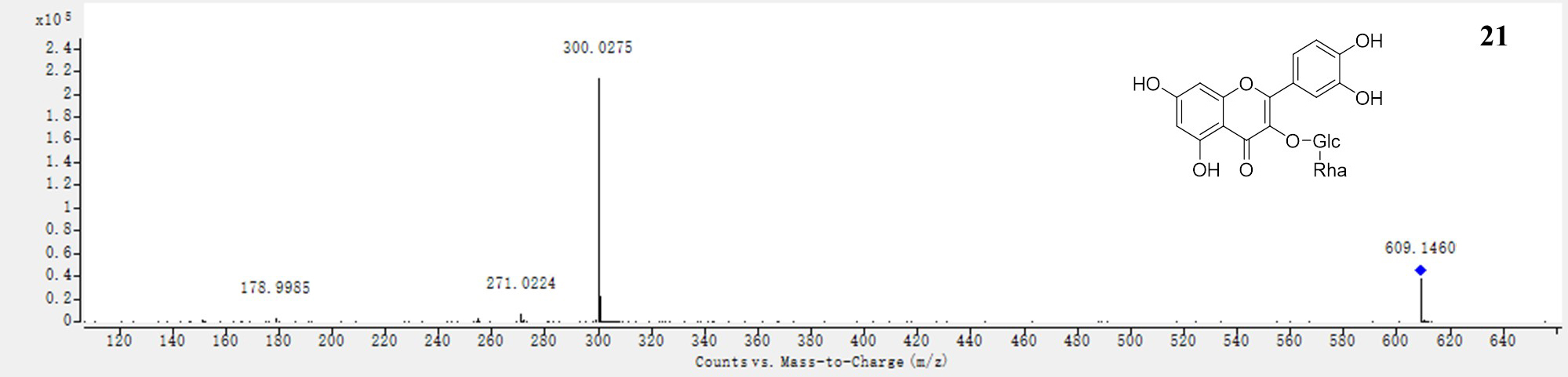


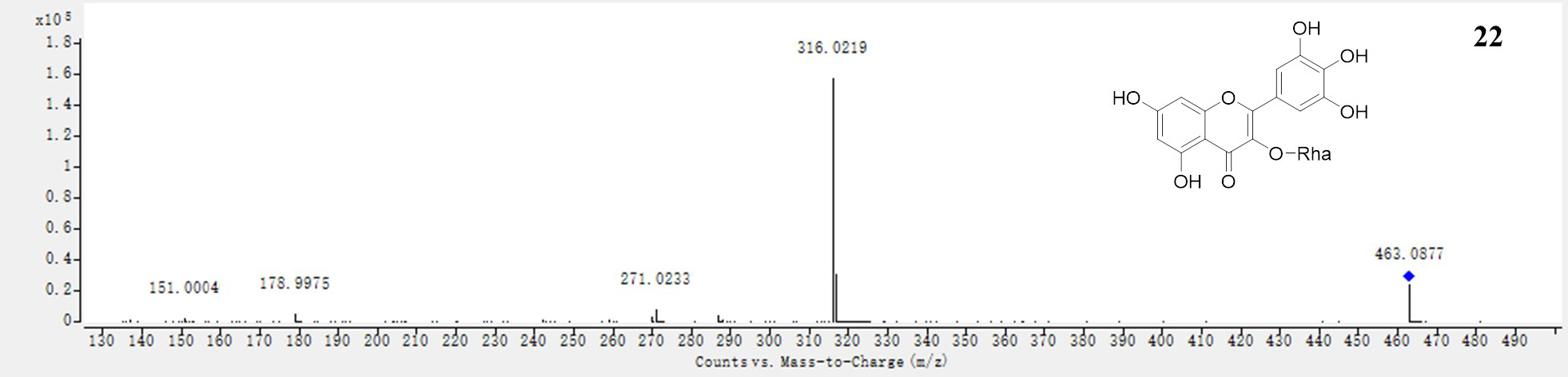


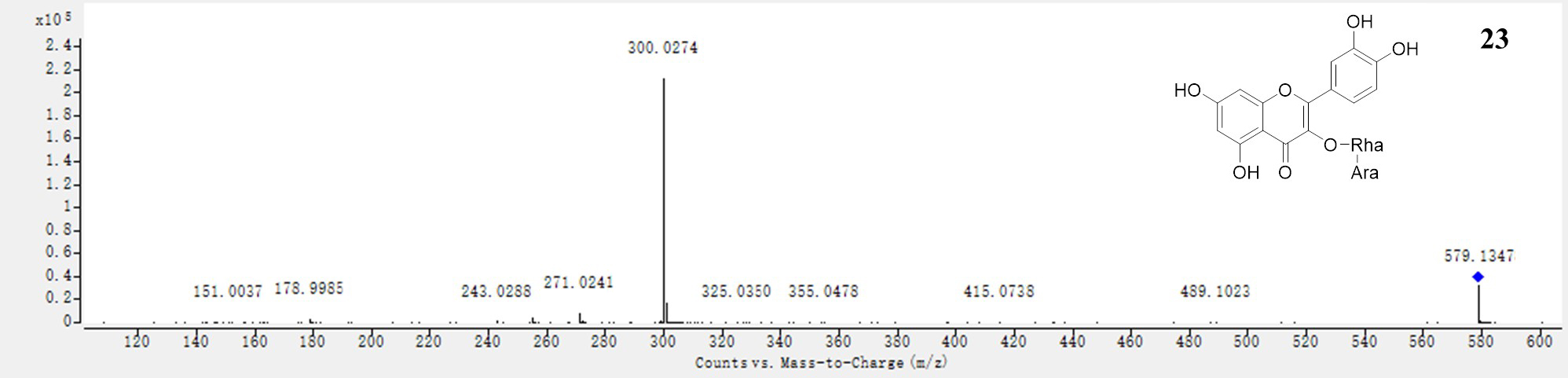


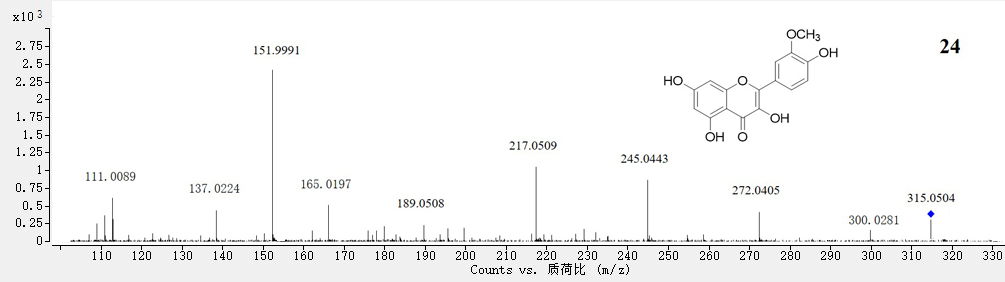


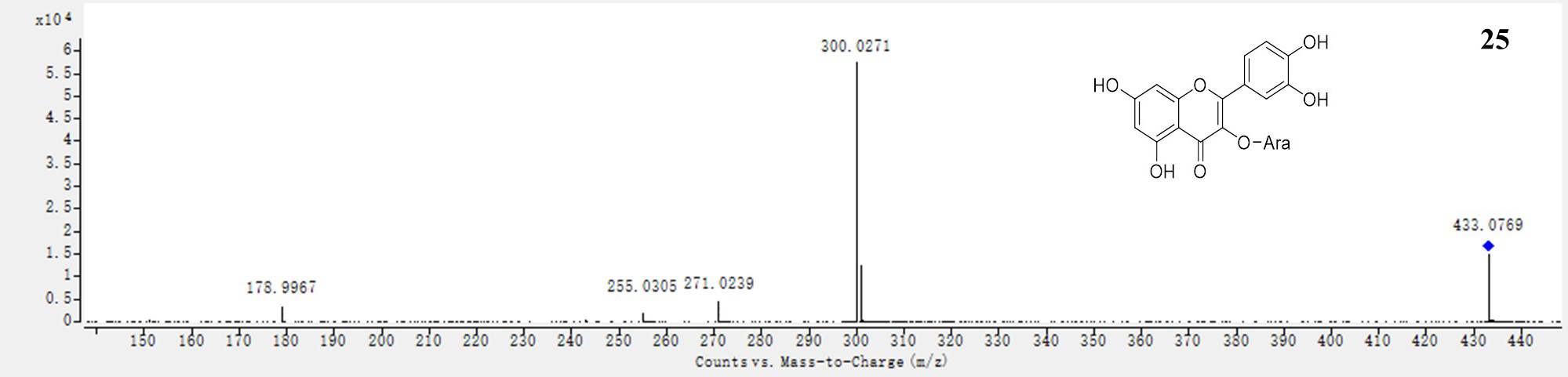


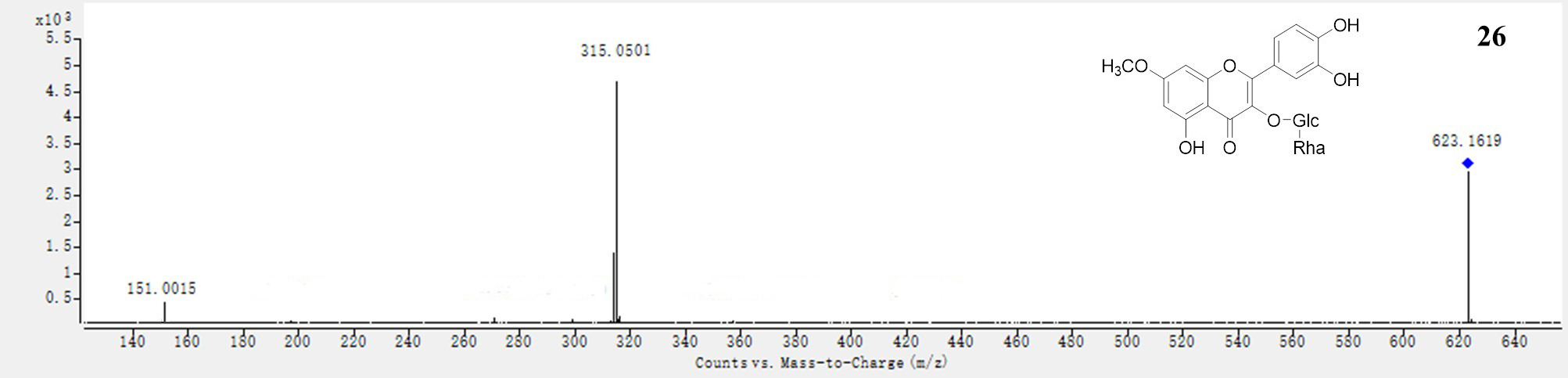


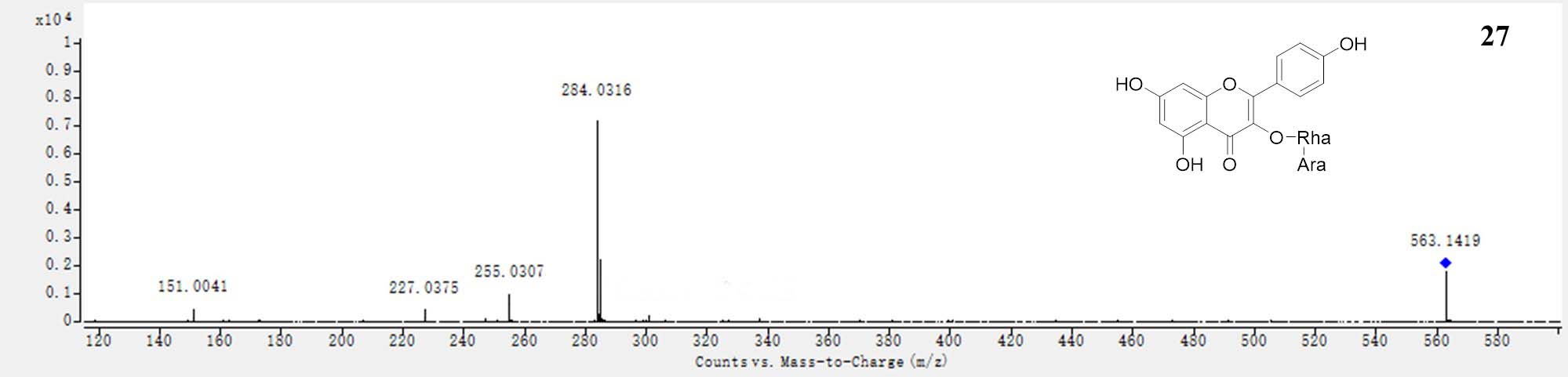


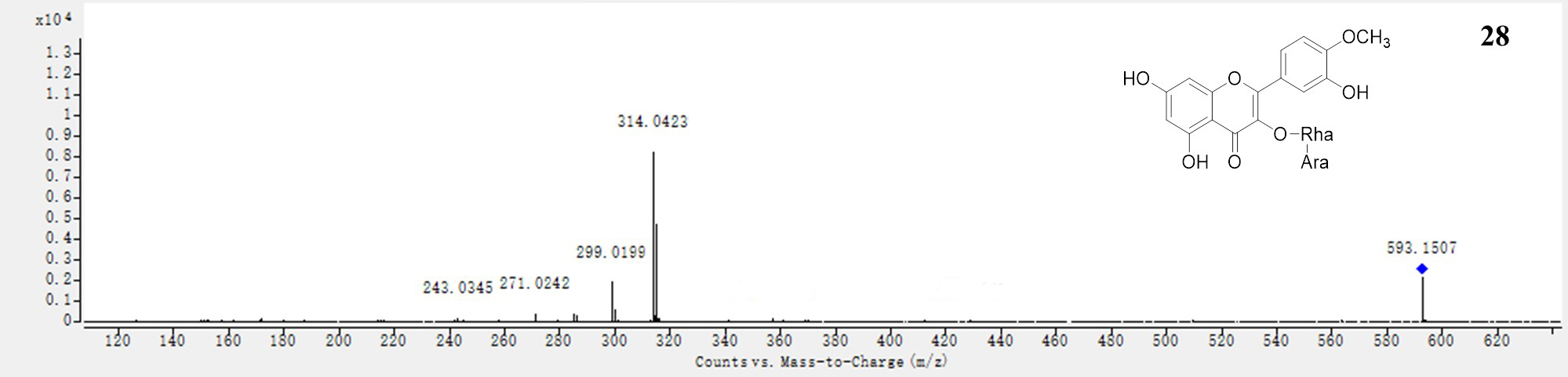


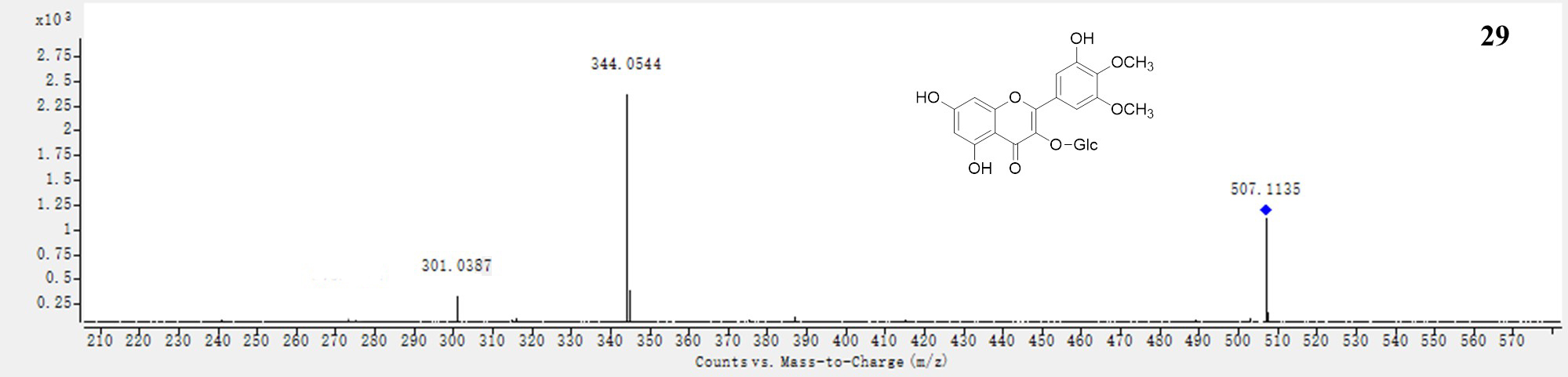


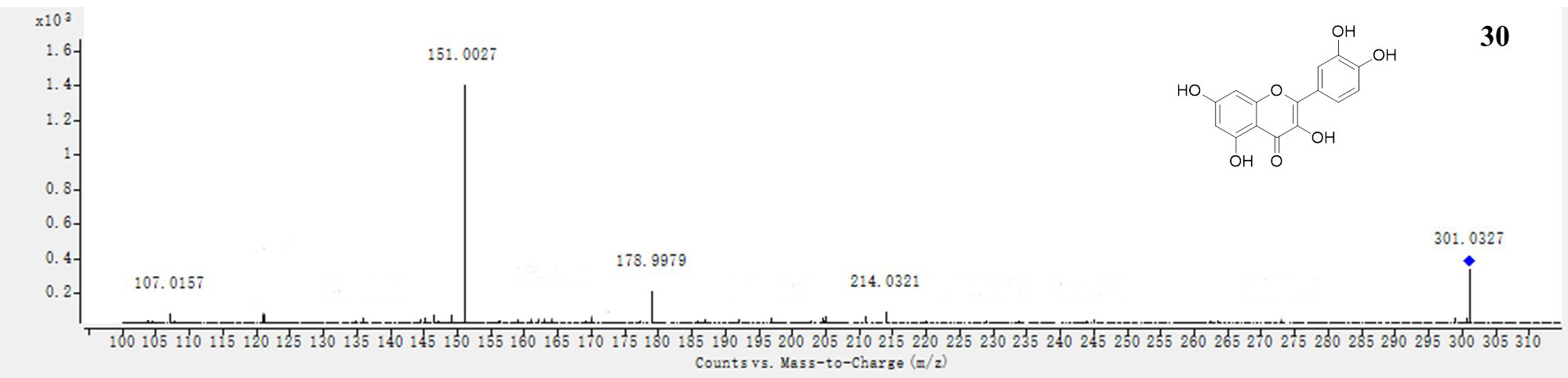


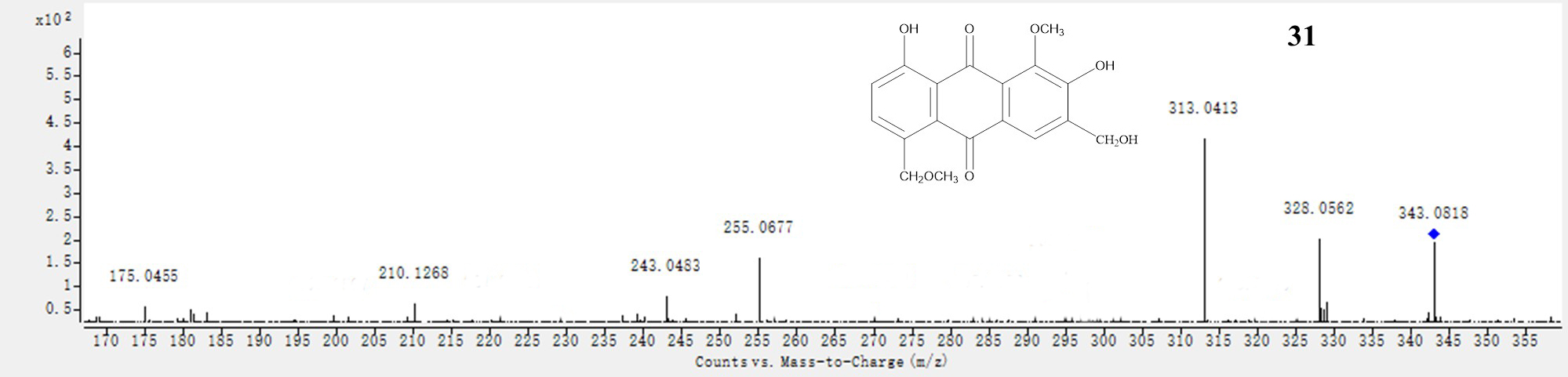


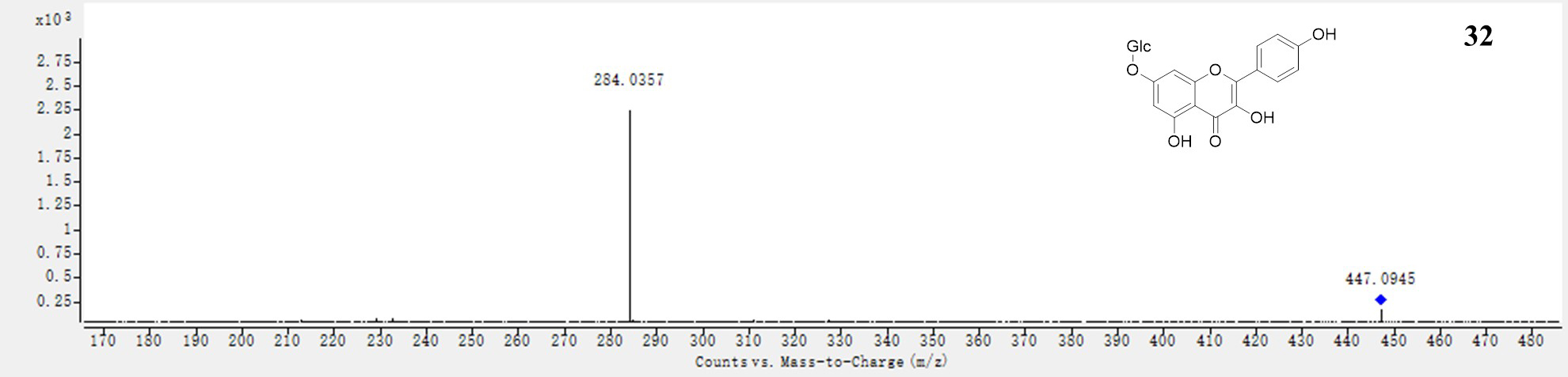


Fig. S3 The ^1^H-NMR (A) and ^13^C-NMR spectra (B) of compound **15**.


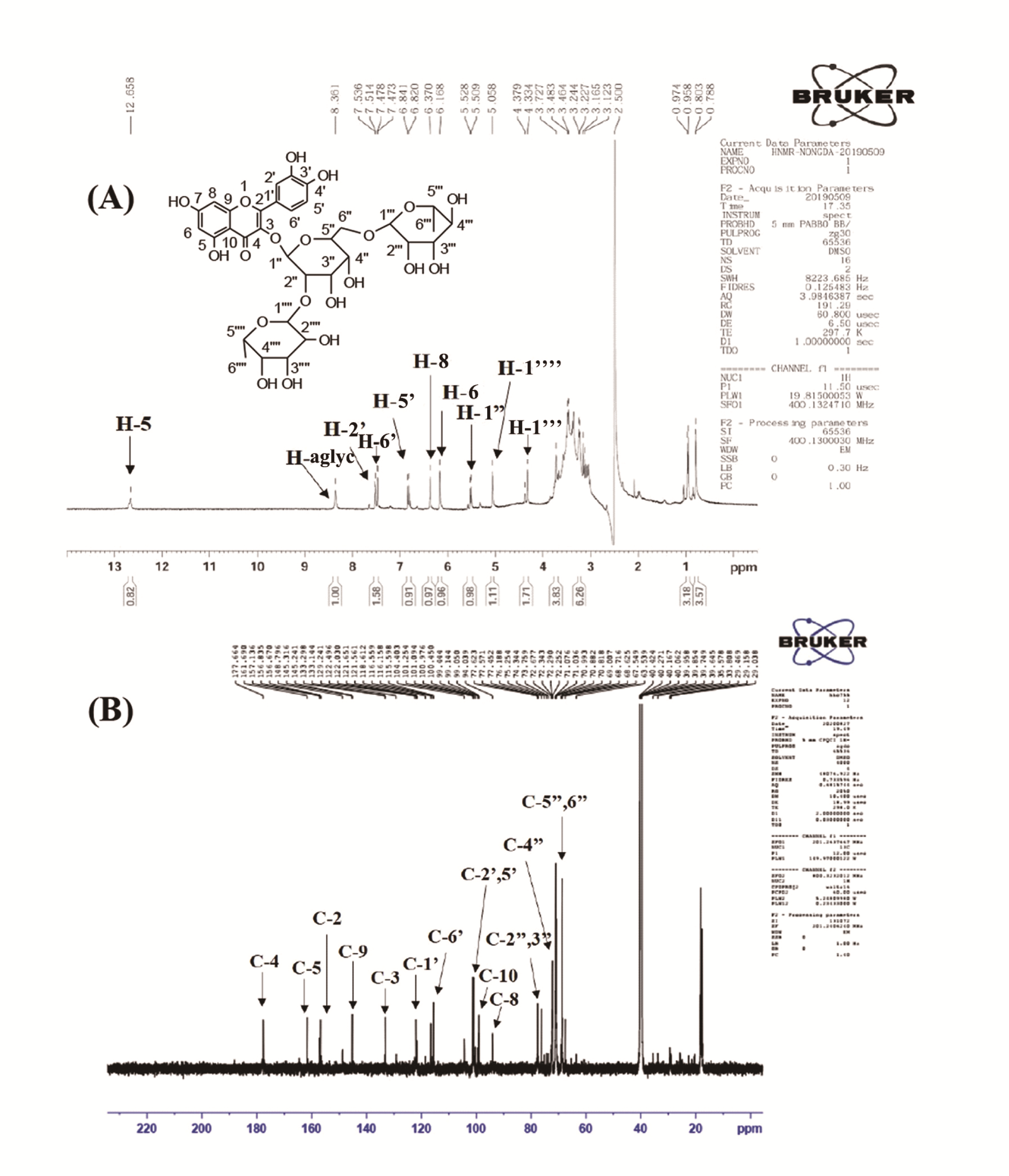


Fig. S4 PCoA analysis of bacterial community compositions in mice gut.


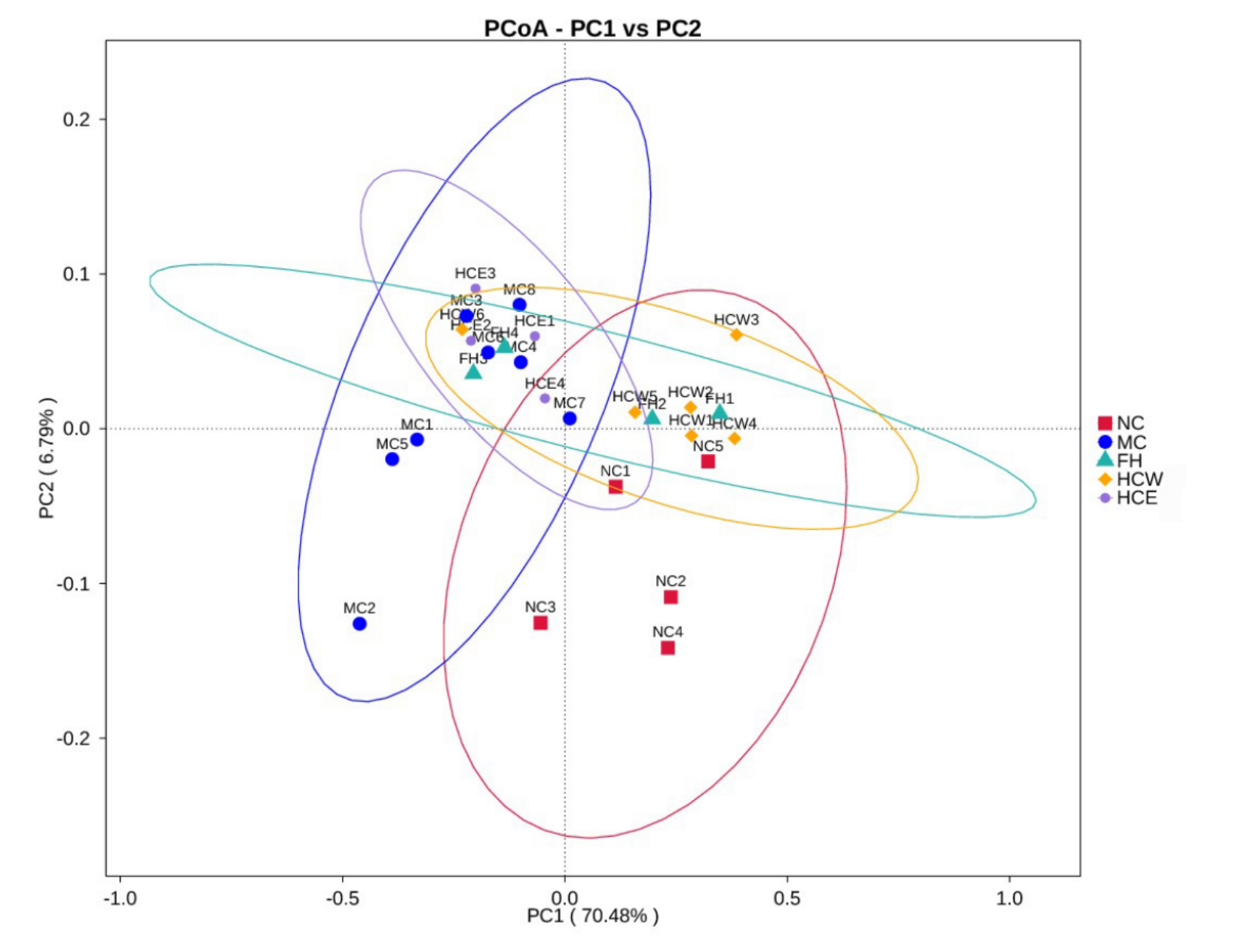


Figure. S5 Reflectance curve (A) and Venn diagram (B) of intestinal microbial OTUs from RT-treated CUMS mice and controls, and the PCoA(C) analysis of bacterial community compositions in mice gut. NC: normal group; MC: model control group; FH: fluoxetine hydrochloride group; RT: rutin group.





Figure. S6 The alpha diversity of the ACE (A), Chao 1 (B), Shannon (C) and Simpson (D) index of intestinal flora. Data are reported as mean ± SD. For statistical significant, ^#^*P* < 0.05 compared with the normal group; **P* < 0.05 compared with the model control group. NC: normal group; MC: model control group; FH: fluoxetine hydrochloride group; RT: rutin group.





Figure. S7 Comparison of the relative abundance of intestinal flora at the phylum (A) and genus level (B).





Fig. S8 The UV spectrum of fraction 2 (compound **15** was isolated by preparative HPLC).


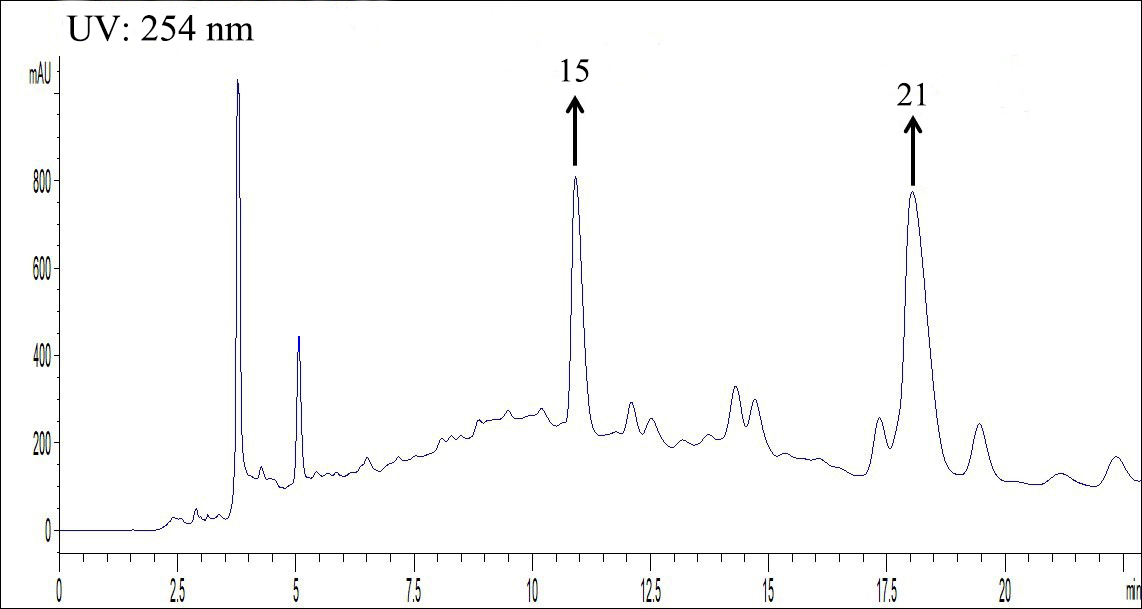


Fig. S9 Schedule of the antidepressant experimental procedures.


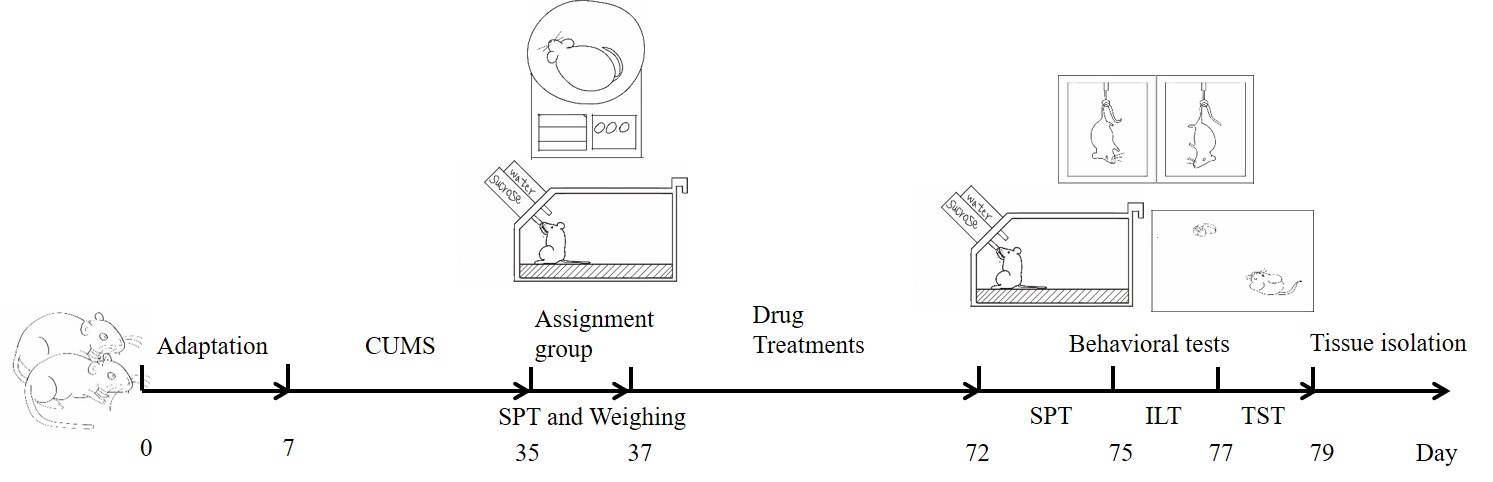


Table S1 Trial groups, number of mice and dose design in the anti-depressive experiments of rutin (**21**)

| Group | Number of Mice | dose  （mg/kg） |
| --- | --- | --- |
| Normal control group (NC) | 10 | -- |
| Model control group (MC) | 10 | -- |
| Positive control group (FH) | 10 | 5.2 |
| RTL | 10 | 0.7*^a^* |
| RTM | 10 | 1.8*^b^* |
| RTH | 10 | 6.3*^c^* |
| RTE | 10 | 10*^d^* |

*^a^* The dose was set based on the content of rutin in the 200 mg HCW; *^b^* The dose was set based on the content of rutin in the 500 mg HCW; *^c^* The dose was set almost four times of 1.8 mg/kg; *^d^* 10 mg/kg was set as the highest dose for rutin.

Table. S2 The schedules for establishment the depression model

| Time | Building methods | Specific schedule |
| --- | --- | --- |
| Monday | Forced swimming 10 min + Strobeflash 12 h + Noise interference 30 min | 8:50 am-9:00 am Forced swimming  9:00 am-9:00 pm Strobeflash  9:00 am -9:30 am Noise interference |
| Tuesday | Empty water bottles 12 h + Food deprivation 12 h + overnight illumination | 8:30 pm-8:30 am Food deprivation  8:30 am-8:30 pm Empty water bottles |
| Wednesday | Physical restraint 12 h + Cage tilt 12 h | 9:30 pm-9:30 am Physical restraint  8:30 am-8:30 pm Cage tilt |
| Thursday | Forced swimming 10 min + Strobeflash 12 h+ Noise interference 30 min | 8:50 am-9:00 am Forced swimming  9:00 am-9:00 pm overnight illumination  9:00 am -9:30 am Noise interference |
| Friday | Food deprivation 12 h + Strobeflash 12 h + Noise interference 30 min | 8:30 am-8:30 pm Food deprivation  9:00 am-9:00 pm Strobeflash  9:00 am -9:30 am Noise interference |
| Saturday | Physical restraint 12 h + Cage tilt 12 h | 9:30 am-9:30 pm Physical restraint  9:30 pm-9:30 am Cage tilt |
| Sunday | Empty water bottles 12 h + Wet cage 12 h + overnight illumination | 8:30 am-8:30 pm Empty water bottles  9:00 pm-9:00 am Wet cage |

Table S3 Trial groups, number of mice and dose design in the anti-depressive experiments of the extracts of *H. citrina* flower buds and flowers.

| Group | Number of Mice | Dose  （mg/kg） |
| --- | --- | --- |
| Normal control group (NC) | 10 | -- |
| Model control group (MC) | 10 | -- |
| Positive control group (FH) | 10 | 5.2 |
| WHCWL | 10 | 200 |
| WHCWH | 10 | 500 |
| WHCEL | 10 | 200 |
| WHCEH | 10 | 500 |
| HCWL | 10 | 200 |
| HCWH | 10 | 500 |
| HCEL | 10 | 200 |
| HCEH | 10 | 500 |

Table S4 Trial groups, number of mice and dose design in the anti-depressive experiments of the extracts of *H. citrina* flowers and dried flower buds.

| Group | Number of Mice | dose  （mg/kg） |
| --- | --- | --- |
| Normal control group (NC) | 10 | -- |
| Model control group (MC) | 10 | -- |
| Positive control group (FH) | 10 | 5.2 |
| HCWL | 10 | 200 |
| HCWH | 10 | 500 |
| DHCWL | 10 | 200 |
| DHCWH | 10 | 500 |
| DHCEL | 10 | 200 |
| DHCEH | 10 | 500 |
